# Pivotal roles of Kupffer cells in the progression and regression of DDC-induced chronic cholangiopathy

**DOI:** 10.1038/s41598-018-24825-x

**Published:** 2018-04-23

**Authors:** Leila Jemail, Masashi Miyao, Hirokazu Kotani, Chihiro Kawai, Hirozo Minami, Hitoshi Abiru, Keiji Tamaki

**Affiliations:** Department of Forensic Medicine, Kyoto University Graduate School of Medicine, Kyoto, Japan

## Abstract

Kupffer cells (KCs) are key players in maintaining tissue homeostasis and are involved in various liver diseases. However, the roles of KCs in the pathogenesis of cholangiopathy are largely unknown. We aimed to investigate the precise roles of KCs in both the progression and regression phases of the 3,5-diethoxycarbonyl-1,4-dihydrocollidine (DDC)-induced cholangiopathy model. In the early phase of DDC-induced cholangiopathy, the number of KCs significantly increased over time. Moreover, KCs were associated with abnormal phenotypic changes in other liver cells, such as hepatocytes, biliary epithelial cells, liver sinusoidal endothelial cells, and hepatic stellate cells. In contrast, KC depletion by clodronate administration suppressed the progression of the disease, and maintained the phenotypes of other cells. In the regression phase, the numbers of KCs significantly decreased, and the cells redifferentiated to their quiescent state. In contrast, KC depletion delayed the recovery of cells by maintaining other liver cells in an active state. These findings suggest that KCs play detrimental roles in the progression phase; however, they are beneficial in the regression phase by mediating interactions between other liver cells. Our data provide new insights into the roles of KCs in the pathogenesis of cholangiopathy.

## Introduction

Kupffer cells (KCs), the liver resident macrophages, are the most abundant macrophage population (approximately 80%) in the body^1^. They usually maintain tissue homeostasis within the physiological range by their primary involvement in the regulation of the innate and adaptive immunity^2^. However, in a chronic liver disease setting, KCs frequently induce excessive inflammatory responses, thus leading to damage and negative repercussions in the liver^3^. In contrast, a study using an acute liver injury model demonstrated that KCs play protective roles, mediating a positive outcome in the progression of the disease^4^. This contradiction, resulting from the use of different mouse models, suggests that the roles of KCs are highly heterogeneous, depending on the stage of the liver disease^5^. Moreover, this shows that studies focusing on the roles of KCs in different phases of liver disease are crucial to elucidate the pathogenesis of these diseases.

In addition to the contrasting functions of KCs, previous reports using different mouse models of liver diseases have described the influence of KCs on the phenotypic states of other liver cells, which are dependent on the stage of the liver disease^6^. Activated KCs are known to induce the activation of hepatic stellate cells (HSCs) into myofibroblasts, which are responsible for fibrotic changes, dysfunction of liver sinusoidal endothelial cells (LSECs), prolongation of hepatocyte injury, and proliferation of abnormal biliary epithelial cells (BECs). To date, few reports have demonstrated the roles of KCs in the different stages of chronic cholangiopathy and their interactions with other liver cells.

Chronic cholangiopathies including primary biliary cholangitis and primary sclerosing cholangitis are cholestatic diseases involving the intrahepatic biliary tree. These diseases have limited therapeutic options and often result in liver cirrhosis. It has been reported that the cholangiocyte and progenitor cell response to inflammation, known as a “ductular reaction”, is a key determinant of disease onset and the progression of chronic cholangiopathies^7^. Recently, Best *et al*. also reported that activated KCs contribute to the aggravation of the inflammatory response during the progression of cholangiopathy, through a causative link with the ductular reaction by using the 3,5-diethoxycarbonyl-1,4-dihydrocollidine (DDC)-induced cholangiopathy model^8^. However, the roles of KCs and their interactions with other liver cells in the different phases of cholangiopathy remain largely unknown. Thus, a better understanding of the roles of KCs and the cellular crosstalk that occurs during the pathogenesis of cholangiopathy may lead to novel therapeutic strategies.

In this study, we aimed to clarify the precise roles of KCs in two different phases, the early inflammatory and regression phases of chronic cholangiopathy, using the DDC model and by depleting macrophages with a clodronate-liposome injection. We here demonstrate that KCs play detrimental roles in the progression of this disease, whereas they play beneficial roles during the regression phase. In addition, the interactions between KCs and other liver cells represent one of the main potential mechanisms for maintaining the balance between these beneficial and detrimental effects.

## Results

### DDC-fed mice develop sclerosing cholangitis over time

To determine chronological changes during the early phase of DDC-induced sclerosing cholangitis, we first performed a pathological analysis of livers from control and DDC-fed mice (Supplementary Fig. S1a). Macroscopic analysis of the livers of DDC-fed mice at 14 days of feeding showed a brownish colour change and hepatomegaly, indicating cholestasis and liver injury (Supplementary Figs S2 and S3a). At 3 days of DDC feeding, the spleen weight/body weight ratio, an indicator of portal hypertension, was significantly increased (Supplementary Fig. S3a). In line with these macroscopic results, the serum analysis also revealed liver injury and cholestasis, as shown by significantly increased levels of aspartate aminotransferase (AST), alanine aminotransferase, alkaline phosphatase, direct-bilirubin, indirect-bilirubin, and bile acid in a time-dependent manner (Supplementary Fig. S4a). To exclude the confounding effects of aging, we compared the control mice at 0 days (8-week-old) with the control mice at 14 days (10-week-old), and confirmed that there was no significant difference between the two control groups for all serological parameters (data not shown).

**Figure 1:**
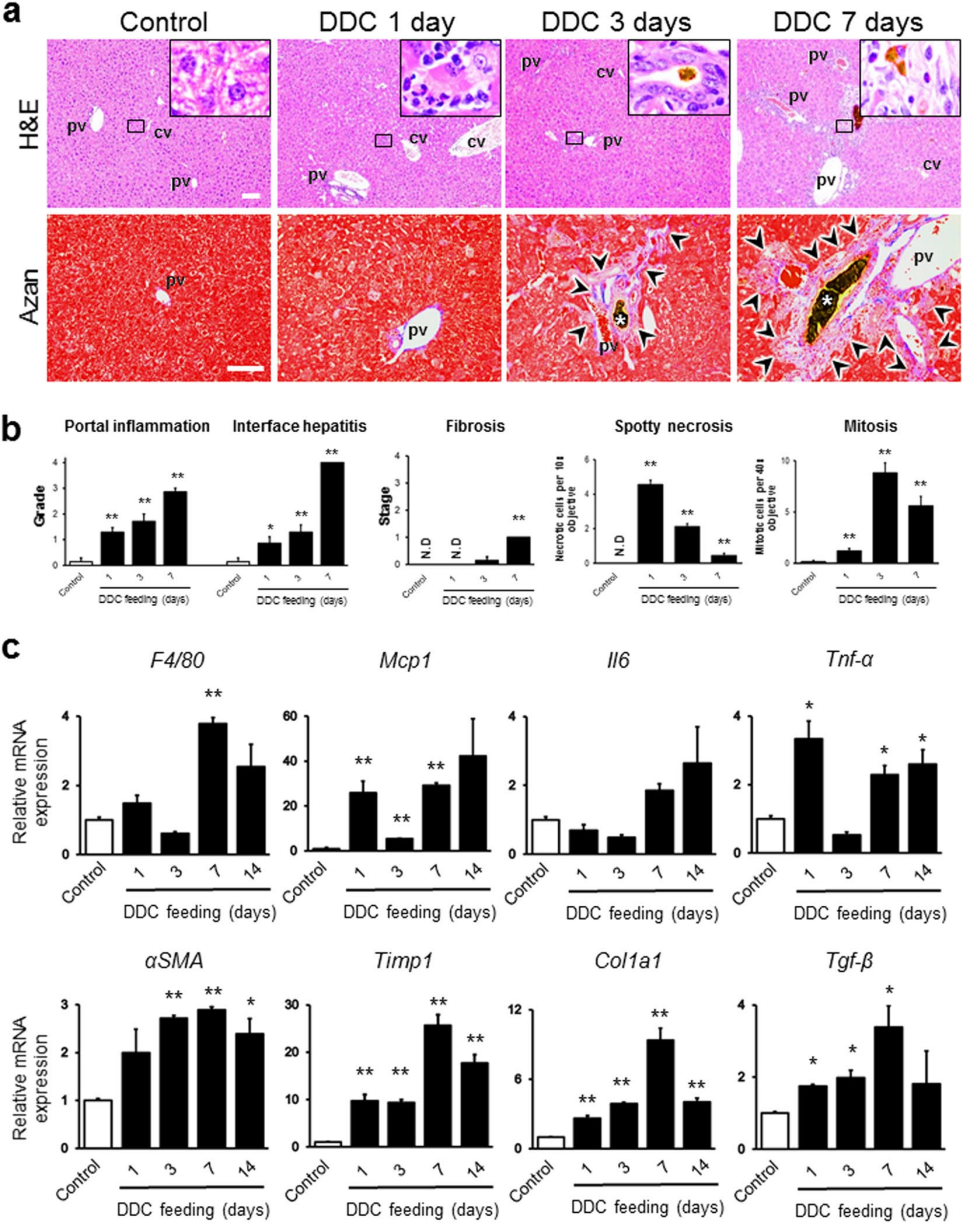
(**a**) Histopathological images of livers from control and 3,5-diethoxycarbonyl-1,4-dihydrocollidine (DDC)-fed mice after 1, 3, and 7 days. Haematoxylin and eosin (H & E) staining (top row) and Azan staining (bottom row) are shown. Bars = 100 μm. The insets of the H&E staining represent high magnification images of the black boxed areas in each group (the control appears normal; the DDC 1-day treatment leads to necrotic hepatocytes with infiltration of inflammatory cells; the DDC 3-day treatment leads to porphyrin plugs in bile ductules; the DDC 7-day treatment leads to fibrotic changes with porphyrin-phagocytosed Kupffer cells around the portal triads). Black arrowheads in the Azan-stained sections highlight the blue-stained fibrotic area. White asterisks in the Azan-stained sections indicate porphyrin plugs. pv, portal vein; cv, central vein. (**b**) Histological grading and staging, using Ishak modified scores (portal inflammation, interface hepatitis, and fibrosis), and number of spotty necroses (per 10× objective) and mitoses (per 40× objective) in random chosen fields in control and DDC-fed mice after 1, 3, and 7 days. Six animals were used in each group. (**c**) Quantitative RT-PCR analysis for proinflammatory genes (F4/80, Mcp1, Il6, and Tnf-α) and profibrogenic genes (αSma, Timp1, Col1a1, and Tgf-β) in control (white boxes) and 3,5-diethoxycarbonyl-1,4-dihydrocollidine (DDC)-fed mice (black boxes) after 1, 3, 7, and 14 days. Values were normalised to GAPDH mRNA expression. Three animals were used in each group. All data are presented as the means ± SEM. *P < 0.05 compared to controls; **P < 0.01 compared to controls; ^#^*P* < 0.05 compared to PBS-injected mice. N.D, not detected.

To further evaluate morphological changes in the early phase of the DDC-induced cholangiopathy model, we performed a histopathological analysis of the DDC-fed mouse livers up to 7 days of feeding. After 1 day of DDC feeding, the haematoxylin and eosin staining of the liver sections showed a singnificant infiltration of inflammatory cells around the portal area, as well as spotty hepatocyte necroses, mainly located around the central vein (Fig. 1a and b). Moreover, the periodic acid Schiff-stained area, indicative of the hepatocyte glycogen storage function, was also significantly reduced compared to that in control mice (Supplementary Fig. S5). Interestingly, the number of spotty hepatocyte necroses peaked at day 1 following DDC feeding; thereafter, their number continued to decrease (Fig. 1a and b). This finding could be explained through compensatory hepatocyte regeneration after 3 days of DDC feeding. In agreement with this finding, hepatocyte mitosis peaked at 3 days of DDC feeding (Fig. 1b). At 3 days of DDC feeding, the inflammatory changes and the loss of glycogen storage were further aggravated, and slight fibrotic changes and porphyrin deposition started to emerge (Figs 1a,b and S5). At 7 days of DDC feeding, a small number of onion skin fibroses (concentric periductal fibrosis), a typical feature of human primary sclerosing cholangitis, was found, as shown by Azan and reticulin staining.

### BECs, HSCs, KCs, and LSECs show altered phenotypes in the DDC-induced progression of cholangiopathy

**Figure 2:**
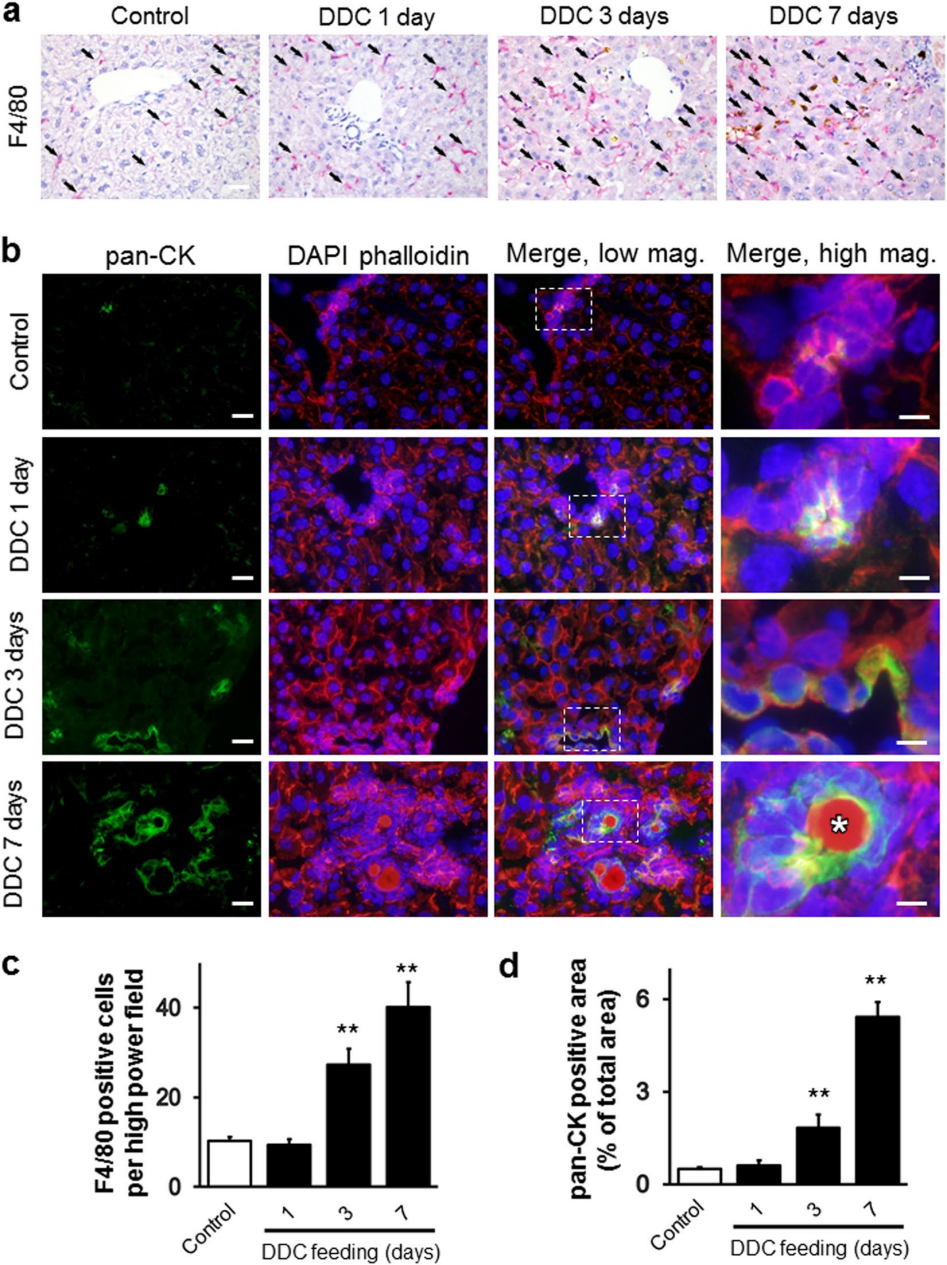
(**a**) Immunohistochemical and immunofluorescent microscopy analyses of livers from control and 3,5-diethoxycarbonyl-1,4-dihydrocollidine (DDC)-fed mice after 1, 3, and 7 days. Liver sections were stained with anti-F4/80 (red) to identify macrophages. Bar = 100 μm. Arrows indicate the cells positive for the antibody. Brown pigments indicate porphyrin accumulation. (**b**) Immunofluorescent microscopy analysis of livers from control and DDC-fed mice after 1, 3, and 7 days. Frozen liver sections were stained with anti-pan-cytokeratin (pan-CK; green) to identify biliary epithelial cells. Nuclei were stained with 4′,6-diamidino-2-phenylindole (DAPI; blue), and the cytoskeleton (F-actin) was stained with phalloidin (red). High-magnification images of the boxed areas, marked with a dotted white line, are shown in the panels on the right. White asterisks highlight the porphyrin plugs in bile ductules. Low-magnification images, bars = 20 μm; high-magnification images, bars = 10 μm. (**c**,**d**) Quantitative analyses for the anti-F4/80-positive cells (**c**) per high power field, and densitometric analysis of pan-CK stained area (**d**) from livers of control (white boxes) and DDC-fed mice (black boxes) after 1, 3, and 7 days. ^**^P < 0.01 compared to controls. N.D., not detected. Ten randomly fields were analysed for each group. Three animals were used in each group.

Increases in the number of BECs, myofibroblasts (mainly composed of activated HSCs), and KCs correlate with the severity of various chronic liver diseases including cholangiopathy^9,10^. Therefore, to determine whether changes in the absolute numbers of BECs, HSCs, and KCs are associated with the DDC-induced progression of cholangiopathy, we next performed a quantitative immunohistochemical analysis of livers from DDC-fed mice up to 7 days of feeding. The main function of HSCs in the perisinusoidal space of the liver (space of Disse) is vitamin A storage through lipid droplets, and maintaining the quiescent state of other liver cells, especially that of LSECs^11^. Under homeostatic conditions, HSCs remain quiescent, storing lipid droplets, but facing liver injury, they are activated and transformed into myofibroblasts, which results in liver fibrogenesis. To evaluate chronological changes of activated HSCs in DDC-induced cholangiopathy, we quantified αSMA-positive myofibroblasts by immunohistochemical examination. Our results showed a significant increase in αSMA-positive cells from day 1 to day 7 of DDC intake (Supplementary Fig. S6a and c). We next examined the gene expression of inflammatory (F4/80, Mcp1, Il6, and Tnf-α) and fibrogenic (αSma, Timp1, Col1a1, and Tgf-β) markers by quantitative RT-PCR analysis to determine functional changes in KCs, hepatocytes, BECs, HSCs, and LSECs in the early phases of the DDC-induced progression of cholangiopathy (Fig. 1c). The mRNA levels of F4/80 and Mcp1, which are macrophage and chemokine markers, respectively, and that of αSma, an HSC activation marker, were significantly increased at around 7 days of DDC feeding, suggesting that the activation of KCs and HSCs occurred around day 7. Furthermore, the levels of Tnf-α, a proinflammatory marker, and those of Timp1, Col1a1, and Tgf-β, which are profibrogenic genes, were elevated from day 1 of DDC intake. Interestingly, the expression of all proinflammatory genes at day 3 was relatively low compared to that in other DDC feeding time periods. In the histological evaluation of chronological changes of KCs and BECs, the colour similarity between the porphyrin pigment and the positive staining of BECs in formalin-fixed paraffin-embedded samples made it difficult to distinguish them from each other (data not shown). Therefore, to detect cells free of porphyrin pigments, we performed immunohistochemical and immunofluorescence analysis using the F4/80 (red colour) and pan-CK marker, indicative of KCs and BEC cells, respectively, in liver sections. As shown in Fig. 2, numbers of both KCs and BECs significantly increased with DDC feeding time. LSECs also represent one of the major constituents of liver non-parenchymal cells. Our SEM analysis showed a loss of fenestrae in LSECs in mice fed DDC for 7 days; such fenestrae are a well-known feature of LSEC injuries^12^ (Supplementary Fig. S7a,b).

### Kupffer cell depletion restores splenomegaly, cholestasis, hepatic glycogen storage, inflammation, and fibrosis in cholangiopathy

To confirm the effect of clodronate liposome administration for macrophage depletion, we repeatedly intraperitoneally injected PBS- or clodronate-liposomes into healthy mice, and analysed their effects by quantitative immunohistochemical examination (Supplementary Fig. S1b). Our results show a significant 89% reduction in liver macrophages in mice which underwent five repeated clodronate injections (Supplementary Fig. S8a,b). The survival difference between the two groups of mice with five repeated injections was not statistically significant based on the log-rank test, and no pathological changes were observed (Supplementary Fig. S8c). These results suggest that this macrophage depletion procedure in itself is not a severe confounding factor for studying the roles of KCs in this experimental setting.

**Figure 3:**
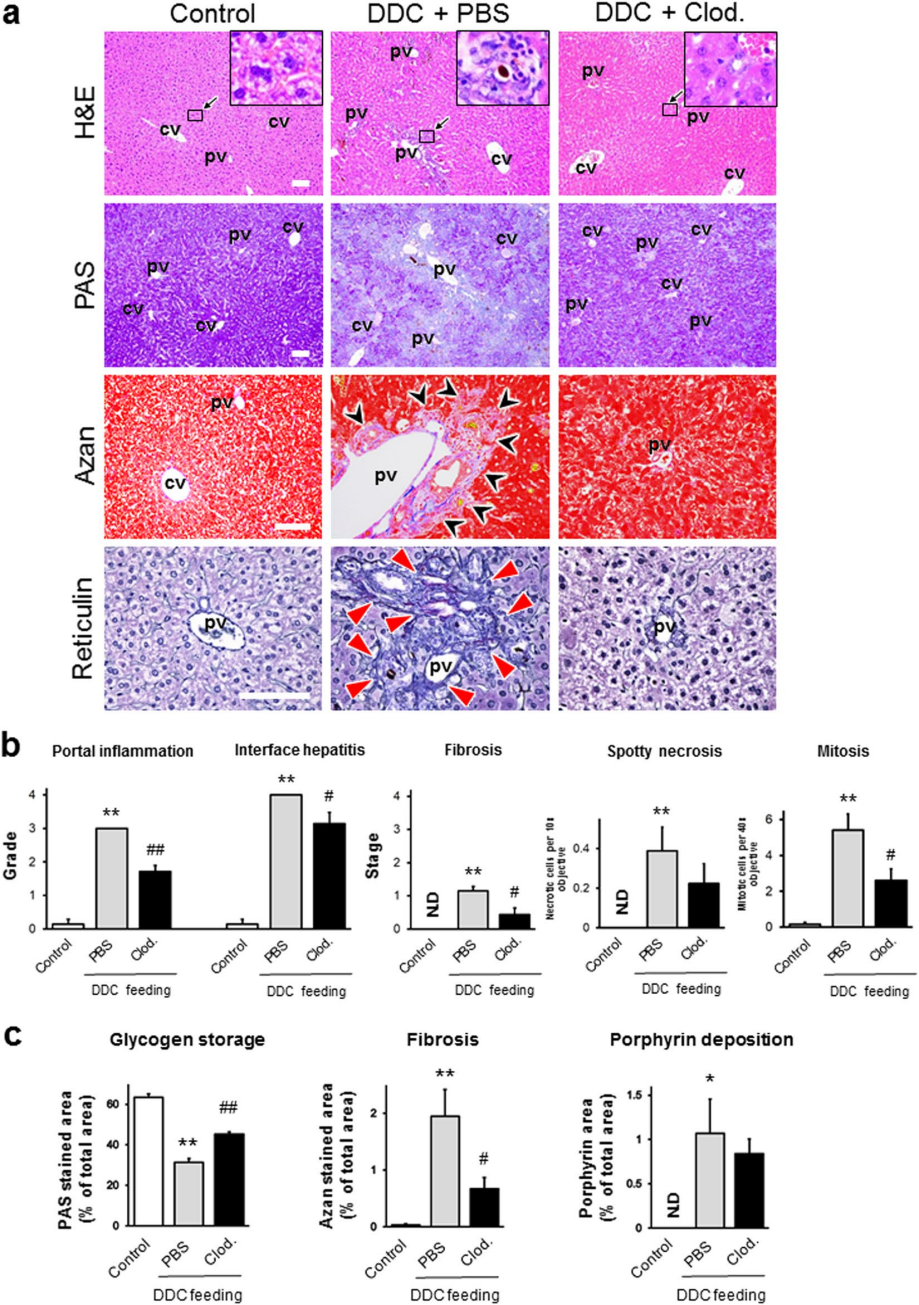
(**a**) Histopathological images of livers from control, PBS-injected, and clodronate (Clod)-injected 7-day 3,5-diethoxycarbonyl-1,4-dihydrocollidine (DDC)-fed mice. Haematoxylin and eosin (H & E) staining (top row), periodic acid Schiff (PAS) staining (second row), Azan staining (third row), and reticulin staining (bottom row) are shown. Bars = 100 μm. The insets of the H&E staining represent high-magnification images of the black-boxed areas in each group (the control appears normal; PBS-injected, DDC-fed mice show porphyrin plugs in bile ductules, with inflammation; clodronate-injected, DDC-fed mice show normal morphology in the interlobular bile duct). Black arrowheads in the Azan staining sections highlight the blue-stained fibrotic area. Red arrowheads in the reticulin staining sections highlight the deposition of immature collagen fibres in portal triads. cv, central vein; pv, portal vein. (**b**) Histological grading and staging using Ishak modified scores (portal inflammation, interface hepatitis, and fibrosis), and the number of spotty necroses (per 10× objective) and mitoses (per 40× objective) in random chosen fields in control, PBS-injected and clodronate (Clod)-injected 7-day DDC-fed mice. (**c**) Quantitative densitometric analysis of the PAS-stained area (glycogen storage area), the blue-stained area for Azan staining (fibrosis area), and brown-stained area for H&E staining (porphyrin deposition area) from livers of control, PBS-injected, and clodronate (Clod)-injected 7-day DDC-fed mice. Data are presented as the means ± SEM. ^*^P < 0.05 compared to controls; ^**^P < 0.01 compared to controls; ^#^*P* < 0.05 compared to PBS-injected mice; ^##^*P* < 0.01 compared to PBS-injected mice. N.D., not detected. Six animals were used in the control group, and five animals were used in the PBS-injected and Clod-injected 7-day DDC-fed groups.

To evaluate the involvement of KCs in macroscopic pathological changes, such as body weight loss, hepatomegaly, and splenomegaly during the progression of cholangiopathy, we compared the weight of the body, liver, and spleen between PBS- and clodronate-injected mice which underwent 7 days of DDC feeding. As shown in Supplementary Fig. S3b, the spleen weight was significantly reduced in clodronate-injected 7-day DDC-fed mice, suggesting that KC depletion suppresses splenomegaly during the progression of cholangiopathy. However, no significant differences were observed in the body and liver weights. To further study whether KC depletion could restore the pathological changes in the liver in cholangiopathy, we compared the histological scores between PBS- and clodronate-injected mice which underwent 7 days of DDC feeding. The excessive enhancement of inflammatory and regenerative responses and the loss of hepatic glycogen storage as a consequence of DDC feeding were significantly suppressed by KC depletion (Fig. 3a–c). The fibrotic changes were slightly but significantly reduced by KC depletion. The numbers of spotty necroses and the levels of porphyrin deposition showed a decreasing tendency in KC-depleted mice. Moreover, the serum levels of cholestasis markers (direct-bilirubin, indirect-bilirubin, and bile acid) were relatively restored in clodronate-injected mice, suggesting that KC depletion ameliorates cholestasis during the progression of cholangiopathy (Supplementary Fig. S4b). Contrary to our expectations, the serum levels of AST, a liver injury marker, following KC depletion in mice fed DDC for 7 days was significantly higher than that in the PBS-injected group.

### Kupffer cell depletion alleviates liver injury in cholangiopathy by preventing activation of BECs, HSCs, and LSECs

**Figure 4:**
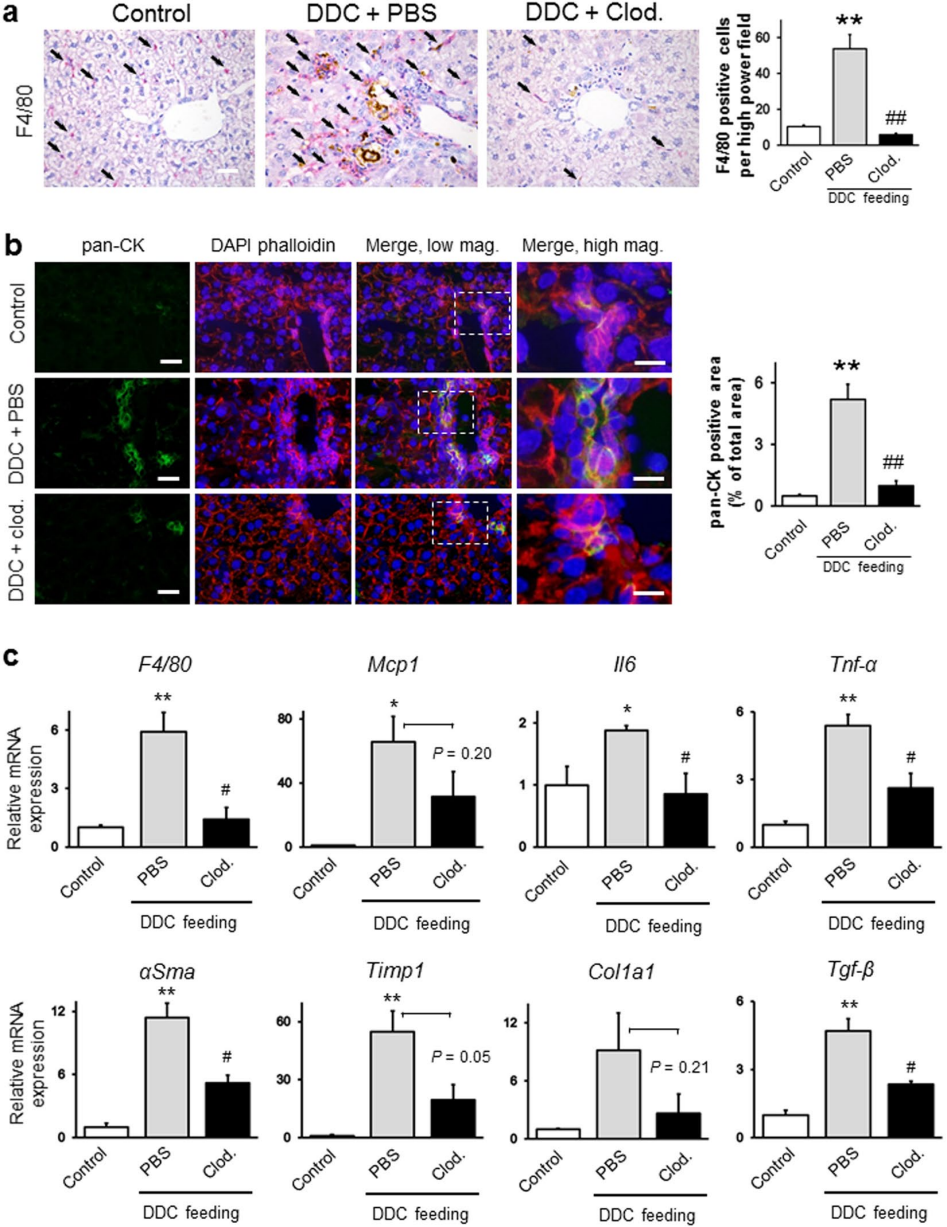
(a) Immunohistochemical analyses of livers from control, PBS-injected and clodronate (Clod)-injected 7-day 3,5-diethoxycarbonyl-1,4-dihydrocollidine (DDC)-fed mice. Liver sections were stained with anti-F4/80 (red) to identify macrophages. Bar = 100 μm. Arrows indicate the cells positive for the antibody. Brown pigments indicate porphyrin accumulation. Quantitative analyses (right graph) for the anti-F4/80-positive cells per high-power field from livers of control (white boxes), PBS-injected (gray boxes), and clodronate-injected (black boxes) 7-day DDC-fed mice. (**b**) Immunofluorescence microscopy analysis of livers from control, PBS-injected and clodronate (Clod)-injected 7-day DDC-fed mice. Frozen liver sections were stained with anti-pan-cytokeratin (pan-CK; green) to identify biliary epithelial cells. Nuclei were stained with 4′,6-diamidino-2-phenylindole (DAPI; blue), and the cytoskeleton was stained with phalloidin (red). High-magnification images of the boxed areas, marked with a dotted white line, are shown in the panels on the right. Low-magnification images, bars = 20 μm; high-magnification images, bars = 10 μm. Quantitative densitometric analysis of the pan-CK-stained area (right graph) from livers of control (white boxes), PBS-injected (gray boxes), and clodronate-injected (black boxes) 7-day DDC-fed mice. Ten randomly chosen fields were analysed for each group. (**c**) Quantitative RT-PCR analysis of proinflammatory genes (F4/80, Mcp1, Il6, and Tnf-α) and profibrogenic genes (αSma, Timp1, Col1a1, and Tgf-β) in control (white boxes), PBS-injected (gray boxes), and clodronate (Clod)-injected (black boxes) 7-day DDC-fed mice. Values were normalised to *GAPDH* mRNA expression. All data are presented as the means ± SEM. ^*^P < 0.05 compared to controls; ^**^P < 0.01 compared to controls; ^#^*P* < 0.05 compared to PBS-injected mice, ^##^*P* < 0.01 compared to PBS-injected mice. Three animals were used in each group.

To determine whether clodronate-liposomes could deplete macrophages not only under healthy conditions, but also during the progression of cholangiopathy, we repeatedly injected PBS- or clodronate-liposomes into mice 2 days before and during the 7 days of DDC feeding, followed by immunohistochemical staining for F4/80 (Supplementary Fig. S1b). As shown in Fig. 4a, the number of F4/80 positive macrophages in clodronate-injected mice was significantly reduced (by 71%) compared to that in PBS-injected mice, confirming the KC depletion effect of clodronate liposomes during the progression of cholangiopathy.

**Figure 5:**
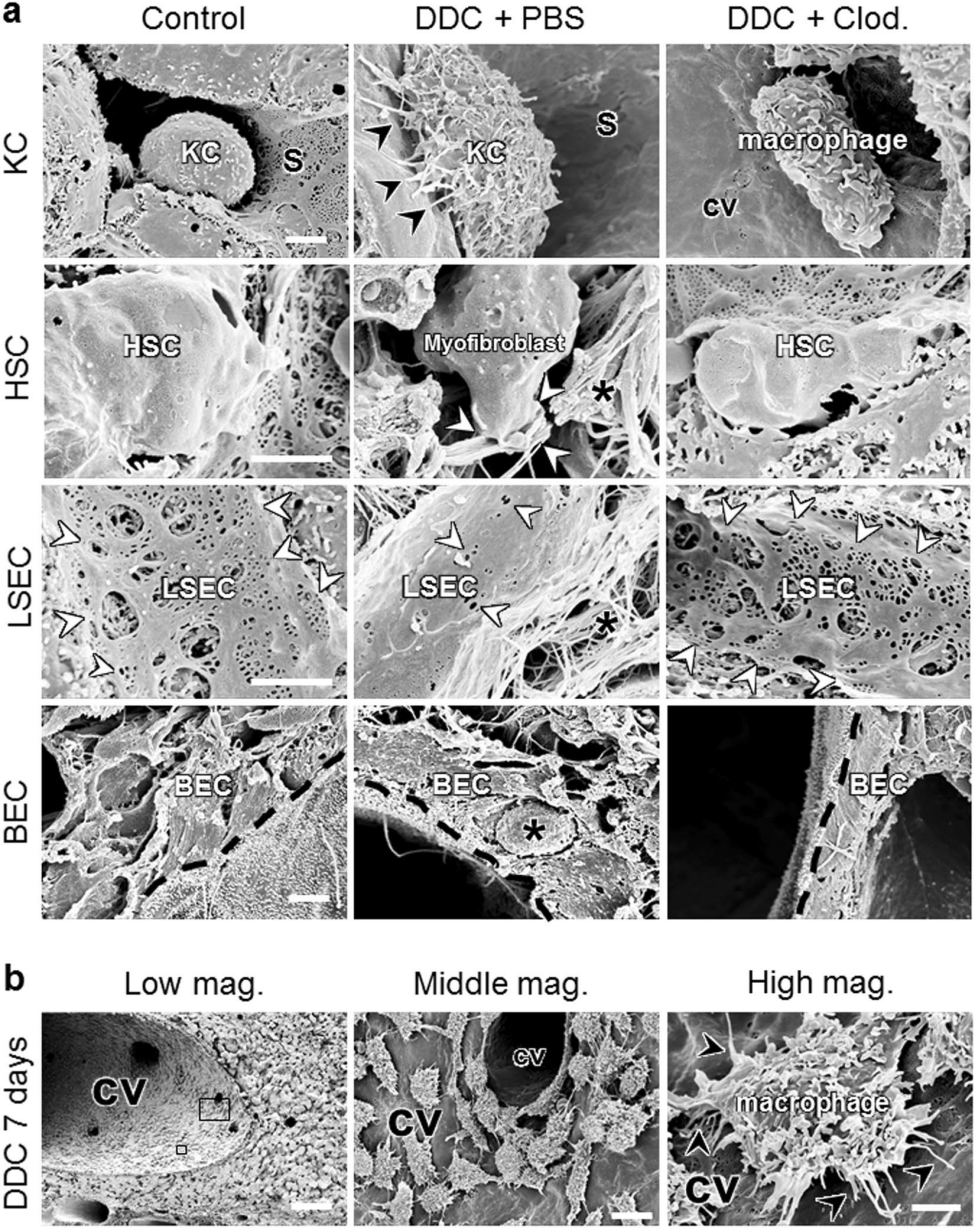
Scanning electron microscopy (SEM) photographs of control, PBS-injected and clodronate-injected 7-day 3,5-diethoxycarbonyl-1,4-dihydrocollidine (DDC)-fed mice. (**a**) Upper panels show Kupffer cells (KCs). Black arrowheads highlight the extending pseudopodia of a KC. The second panel shows hepatic stellate cells (HSCs). White arrowheads highlight collagen fibres secreted by myofibroblasts. The black asterisk shows excessive collagen deposition. The third panel shows liver sinusoidal endothelial cells (LSECs). White arrowheads show fenestrae and gaps in LSECs. Bottom panels show biliary epithelial cells (BECs). Black dotted lines highlight the lumens of the bile ducts. The black asterisk shows infiltration of inflammatory cells between two BECs. Bars = 2 μm. (**b**) SEM photographs of a 7-day DDC-fed mouse. The left panel shows a low-magnification image (bar = 100 μm). A big boxed area highlights an intermediate-magnification image (middle panel; bar = 10 μm). A small boxed area highlights a high-magnification image (right panel; bar = 2 μm). Black arrowheads indicate extending pseudopodia of a KC. cv, central vein; S, sinusoid lumen. Three animals were used in each group.

To examine whether KCs could contribute to the differentiation of BECs and HSCs during the progression of cholangiopathy, we performed an immunohistochemical analysis of pan-CK and αSMA in PBS- and clodronate-injected 7-day DDC-fed mice. The pan-CK areas and αSMA-positive cells in the livers of clodronate-injected mice were significantly reduced by 81% and 56%, respectively, indicating either indirect activation of BECs and HSCs by KCs via hepatocyte damage and cholestasis or direct activation of these cells in disease progression (Figs 4b and S6b and d). In line with these morphological analyses, the mRNA expression of proinflammatory and profibrogenic genes showed 2- to 3-fold decrease in clodronate-injected mice fed DDC for 7 days (Fig. 4c). To clarify whether KCs could also be responsible for the differentiation of LSECs, we evaluated porosity in PBS- and clodronate-injected mice fed DDC for 7 days by SEM. The severity of the decrease in porosity following DDC intoxication was delayed by 56% in clodronate-injected mice, indicating that KCs cause an unfavourable activation of LSECs (Figs 5a and S7a and c)^13^.

These results suggest that KCs could be essential for activation of BECs, HSCs, and LSECs, and a major determinant of inflammatory and fibrogenic responses during the progression of cholangiopathy.

### Kupffer cell depletion results in amelioration of ultrastructural and organellar injury during the progression of cholangiopathy

Evaluation of lipid storage in the liver using formalin-fixed paraffin-embedded samples is difficult because lipids are dissolved during the preparation of the sample. Thus, to evaluate whether KCs could influence lipid storage in the liver during the progression of cholangiopathy, we next performed toluidine blue staining for semithin liver sections of resin-embedded specimens, which represents a suitable evaluation method for lipid storage. As shown in Supplementary Fig. S9, DDC feeding induced an extreme increase in fat-laden macrophages, with giant cell formation in the extravascular area, suggesting the activation of KCs. In addition, quiescent HSCs, fat-storing cells in the Disse space, were almost abolished by DDC intoxication. Instead, spindle-shaped myofibroblasts emerged especially around necrotic hepatocytes, suggesting the activation of HSCs. In contrast, the depletion of KCs by clodronate in DDC-fed mice inhibited the activation of KCs and HSCs.

**Figure 6:**
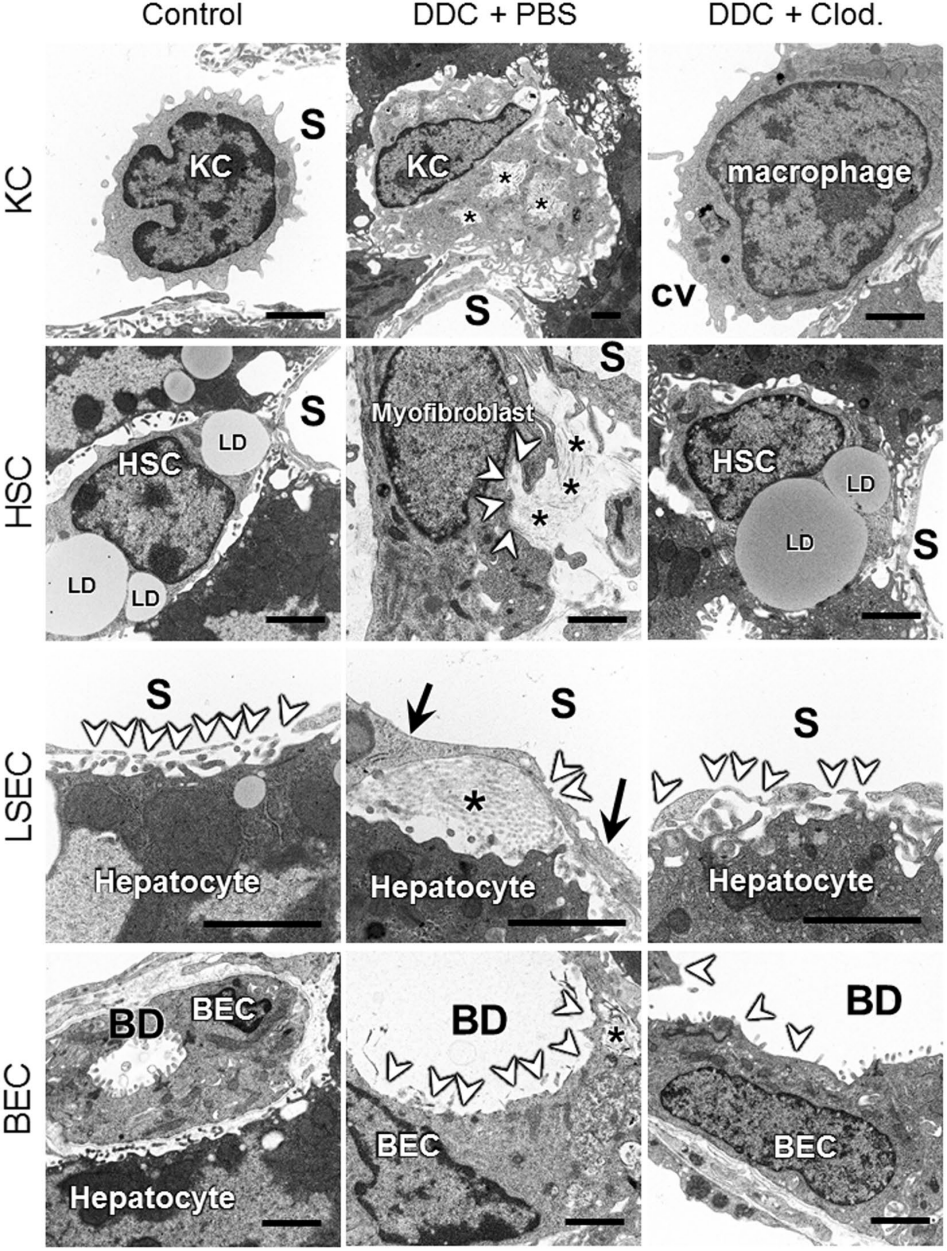
Transmission electron microscopy photographs of control, PBS-injected and clodronate-injected 7-day 3,5-diethoxycarbonyl-1,4-dihydrocollidine (DDC)-fed mice. Upper panels show Kupffer cells (KCs). Black asterisks in the middle panel show porphyrin crystals phagocytosed by a KC. The second panel shows hepatic stellate cells (HSCs). White arrowheads highlight collagen fibres secreted by myofibroblasts. Black asterisks show collagen fibres. The third panel shows liver sinusoidal endothelial cells (LSECs). White arrowheads show fenestrae in LSECs. Black arrows highlight a thickened endothelial wall. The black asterisk indicates collagen fibre deposition. The bottom panels show biliary epithelial cells (BECs). The white arrowheads highlight the loss of microvilli in BECs. Bars = 2 μm. BD, bile duct lumen; cv, central vein; LD, lipid droplet; S, sinusoid lumen. Three animals were used in each group.

To evaluate the roles of KCs in cholangiopathy at the ultrastructural level, we next performed SEM and TEM analyses in PBS- and clodronate-injected mice fed DDC for 7 days. DDC feeding triggered KC activation, as evidenced by an extension of pseudopodia (Fig. 5a), with active phagocytosis of porphyrin crystals (Fig. 6). In contrast, injection of clodronate led to markedly reduced numbers of intralobular macrophages, and to a decrease in the activation of remaining macrophages (Figs 5a and 6). Intoxication with DDC resulted in a large accumulation of macrophages in the large central veins (Figs 5b and S10). The presence of macrophages in the large central veins was not abolished by the injection of clodronate (data not shown). These results suggest that clodronate predominantly depletes KCs (intralobular macrophages), rather than recruited monocytes (macrophages in the central vein)^5^. In PBS-injected DDC-fed mice, other liver cells were also activated following KC activation. Thus, HSCs lost their initial quiescent state and function and converted into myofibroblasts secreting collagen fibres. BECs assumed a reactive state, with neutrophil infiltration, loss of microvilli, and intracellular porphyrin crystal injuries. LSECs lost their fenestrae and the cell wall thickened, with an accumulation of collagen fibres. Hepatocytes became necrotic or showed altered architecture, including bile canalicular disorganisations (Supplementary Fig. S11). In addition, DDC feeding also impaired hepatocyte organelles, as evidenced by deformed mitochondria with disarranged cristae, abnormal elongated endoplasmic reticula (ER), decreased numbers of ribosomes and glycogen particles, and cytoskeletal dysfunction, as shown by bleb formations in the hepatocellular (bile canalicular) wall (Supplementary Fig. S12). All of these detrimental ultrastructural changes and hepatocyte organelle injuries were improved by clodronate injection, indicating that KCs indirectly induced the hepatocyte damage through inflammation and cholestasis or directly induced hepatocyte injury during disease progression (Supplementary Figs S11 and S12).

### Kupffer cell depletion adversely affects the liver during the regression phase of cholangiopathy

**Figure 7:**
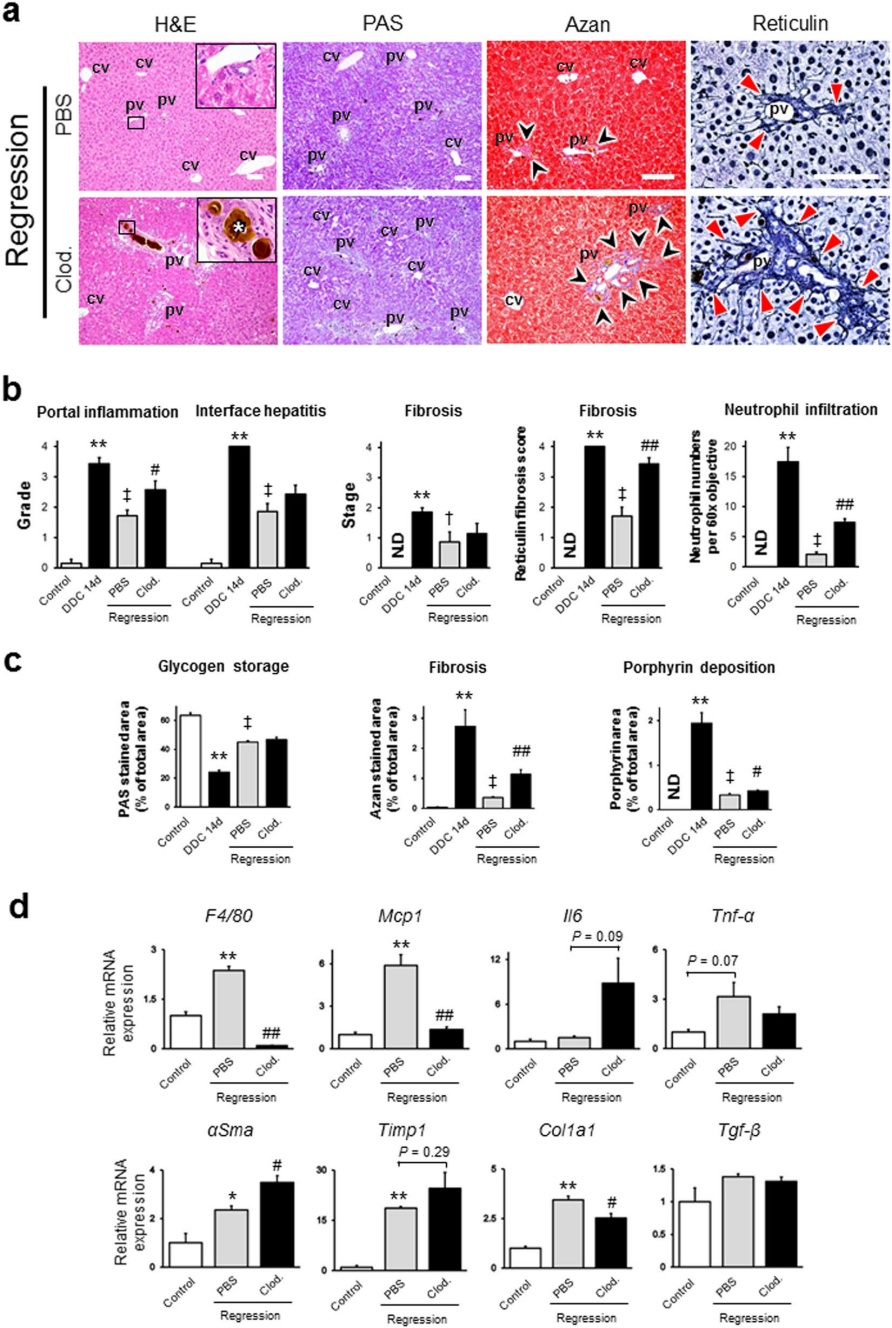
(**a**) Histopathological images of livers from PBS-injected and clodronate (Clod)-injected 7-day regression mice [14 days of 3,5-diethoxycarbonyl-1,4-dihydrocollidine (DDC) feeding, followed by an additional 7 days of standard diet]. Haematoxylin and eosin (H&E) staining, Periodic acid Schiff (PAS) staining, Azan staining, and reticulin staining are shown. The insets of the H&E staining represent high-magnification images of the black-boxed area in each group. White asterisks show porphyrin plugs in bile ductules. Black arrowheads in the Azan-stained sections highlight the blue-stained fibrotic area. Red arrowheads in the reticulin-stained sections highlight the deposition of immature collagen fibres in portal triads. Bars = 100 μm. cv, central vein; pv, portal vein. (**b**) Histological grading and staging using Ishak modified scores (portal inflammation, interface hepatitis, and fibrosis), reticulin fibrosis scores, and the numbers of infiltrating neutrophils per 60× objective in randomly chosen fields in PBS-injected and clodronate-injected 7-day regression mice. (**c**) Quantitative densitometric analysis of the PAS-stained area (glycogen storage area), the blue-stained area in Azan staining (fibrosis area), and the brown-stained area in H & E staining (porphyrin deposition area) from livers of PBS-injected and clodronate-injected 7-day regression mice. Six animals were used in the control group, and five animals were used in the PBS-injected and Clod-injected 7-day regression groups. (**d**) Quantitative RT-PCR analysis for proinflammatory genes (F4/80, Mcp1, Il6, and Tnf-α) and profibrogenic genes (αSma, Timp1, Col1a1, and Tgf-β) in PBS-injected and clodronate-injected 7-day regression mice. Values were normalised to *GAPDH* mRNA expression. Three animals were used in each group. All data are presented as the means ± SEM. ^*^P < 0.05 compared to controls; ^**^P < 0.01 compared to controls; ^†^*P* < 0.05 compared to 14-day DDC-fed mice; ^‡^*P* < 0.01 compared to 14-day DDC-fed mice; ^#^*P* < 0.05 compared to PBS-injected, 7-day regression mice; ^##^*P* < 0.01 compared to PBS-injected, 7-day regression mice. N.D., not detected.

Previous studies have described that macrophages play critical roles in tissue repair, by clearing apoptotic cells and debris, promoting epithelial repair, antagonizing proinflammatory macrophages, and degrading the extracellular matrix^14^. Therefore, we hypothesised that KCs promote tissue repair during the regression phase of cholangiopathy, and that depletion of KCs delays in recovery. First, to determine the extent of liver recovery following withdrawal of DDC, we compared 14-day DDC-fed mice and 7-day PBS-injected mice in regression after 14 days of DDC feeding (Figs 7a–c, S1c and S13). Our histological analysis in the 7-day regression mice showed a substantial decrease in the infiltration of inflammatory cells, especially in the infiltration of neutrophils. In addition, a significant decline in collagen and reticulin fibres and porphyrin deposition, as characterised by Azan-, reticulin-, and H&E-stained sections, was apparent in 7-day regression mice. Moreover, we observed a significant increase in glycogen storage in the 7-day regression mice, indicating that hepatocyte regeneration could lead to a restoration of hepatic function, following the cessation of DDC intoxication. Consistent with our prediction, we found that some aspects of cholangiopathy in 7-day clodronate-injected regression mice had actually worsened, including the levels of portal inflammation and reticulin fibrosis scores, neutrophil infiltration, and the significantly higher fractions of Azan-stained and porphyrin deposition areas. Additionally, glycogen storage, spotty necrosis, and mitosis levels were not significantly different between the PBS- and clodronate-injected mice following 7 days of recovery. Moreover, the mRNA levels of F4/80 and Mcp1 in clodronate-injected 7-day recovery mice were significantly lower compared to those in PBS-injected mice (Fig. 7d). In line with our histological analysis, the levels of Il6, αSma, and Timp1 in clodronate-injected 7-day recovery mice remained high compared to those in PBS-injected mice. However, contrary to our expectation, the Col1a1 mRNA levels in clodronate-injected 7-day recovery mice were significantly lower than those in PBS-injected mice.

## Discussion

**Figure 8:**
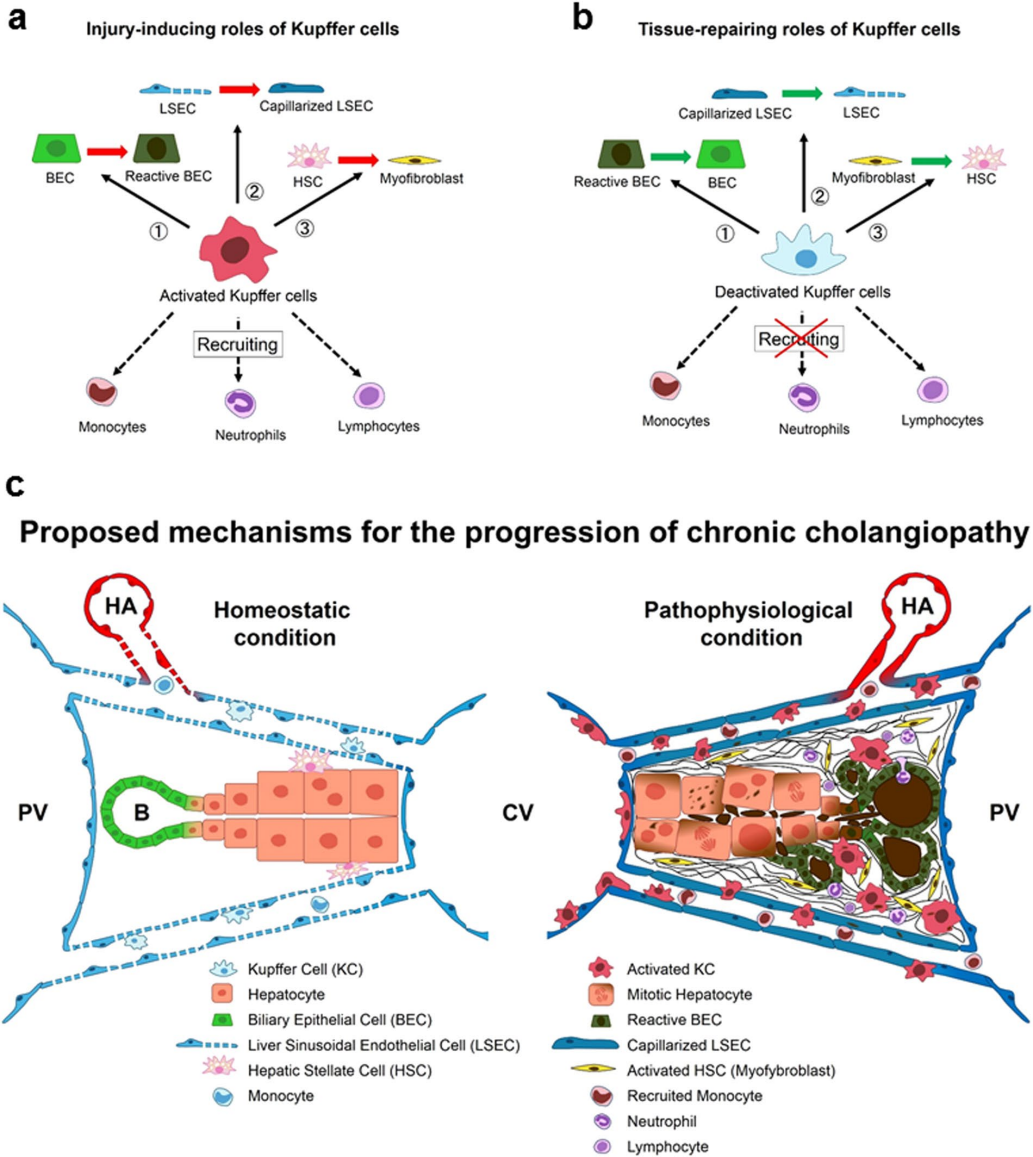
Schemes of (**a**) injury-inducing roles of Kupffer cells (KCs) in the progression phase of cholangiopathy, (**b**) tissue-repairing roles of KCs in the regression phase of cholangiopathy, and (**c**) proposed mechanisms for the progression of cholangiopathy. (**a**) In the early inflammatory phase of cholangiopathy, KCs exert detrimental effects though their proinflammatory changes. The KCs induce (1) activation of quiescent biliary epithelial cells (BECs), resulting in reactive BECs, (2) capillarisation of liver sinusoidal endothelial cells (LSECs) from quiescent LSECs, and (3) activation of quiescent hepatic stellate cells (HSCs) into collagen producing myofibroblasts. The KCs also contribute to the recruitment of proinflammatory monocytes, neutrophils, and lymphocytes. (**b**) In the regression phase of cholangiopathy, KCs tend to have beneficial effects through their anti-inflammatory changes. The KCs induce redifferentiation of abnormally activated (1) BECs, (2) LSECs, and (3) HSCs. The anti-inflammatory KCs suppress the recruitment of inflammatory monocytes, neutrophils, and lymphocytes. (**c**) Under homeostatic conditions (left side of the scheme), quiescent KCs maintain a balance between the proliferation of parenchymal cells and the elimination of apoptotic and/or senescent cells (hepatocytes and BECs) by mediating the secretion of various growth factors and cytokines, and by changing their phagocytic ability. Quiescent KCs, mainly composed of anti-inflammatory (regulatory and wound-healing) macrophages, directly and indirectly preserve the quiescence state in LSECs and HSCs by mediating the secretion of various growth factors, promoting appropriate immune tolerance, and inducing apoptosis of cells that have undergone excessive activation. KCs also contribute to elimination of hydrophobic cytotoxic bile. KCs appropriately change their proliferation activity and recruit bone marrow-derived monocytes. In contrast, under pathophysiological conditions (right side of the scheme), activated KCs, mainly composed of inflammatory macrophages, dysregulate the activity of other liver cells by inducing the unnecessary deaths of parenchymal cells and cholestatic injuries by hydrophobic cytotoxic bile accumulation, and causing abnormal regeneration (dysregulated hepatocyte architecture and reactive BECs). Activated KCs also cause the impairment of non-parenchymal cells, capillarised LSECs and myofibroblasts, by inducing the expression of inflammatory and fibrogenic genes, and disrupting the immune tolerance. Activated KCs contribute to the further proliferation and recruitment of bone marrow-derived monocytes, eventually resulting in vicious cycles of chronic cholangiopathy.

The present study shows that KCs play pivotal roles in the pathogenesis of chronic cholangiopathy in mice (Fig. 8). KCs contributed to the initiation and development of liver injury in the progression phase and, conversely, to the promotion of recovery in the regression phase. In the early phase of progression, KCs activated as a result of DDC intoxication initiated inflammatory responses by secreting inflammatory cytokines and chemokines, leading to activation of BECs, LSECs, and HSCs, and recruiting inflammatory monocytes, neutrophils, and lymphocytes (Fig. 8a). These proinflammatory responses eventually resulted in hepatocyte necrosis, cholestasis, and parenchymal fibrosis. In the disease regression phase, KCs were required to suppress the inflammatory and fibrogenic changes, in contrast to the progression phase (Fig. 8b). Deactivated KCs in the regression phase may contribute to the inactivation and redifferentiation of BECs, LSECs, and HSCs (Fig. 7d). Deactivated KCs could also suppress the recruitment of these cells to the liver, as shown by significantly higher levels of portal inflammatory cell infiltration in KC-depleted mice (Fig. 7a and b)^15^. These findings indicate that KCs play dual roles in cholangiopathy: a detrimental role during the progression phase and a beneficial role during the regression phase. We thus propose a KC-mediated mechanism during the progression of cholangiopathy: KCs generally maintain homeostasis by their immunomodulatory and phagocytic effects, whereas during the progression of cholangiopathy, they have detrimental effects, such as induction of excessive inflammatory, cholestatic, and fibrogenic responses (Fig. 8c). Therefore, the roles of KCs could be dependent on the phase of the liver disease.

We propose that focusing on the phase of liver disease is essential to clarify the disease pathogenesis, especially in chronic liver diseases. We have thus far demonstrated the emergence of pathological changes during the early phase of chronic liver diseases using animal models, our current model, and NAFLD/NASH models^13,16^. The DDC diet model used in the current study is feasible to analyse the detailed chronology of cholangiopathy because the model progresses slowly enough to provide time for evaluation of the pathophysiological changes occurring in each phase. In addition, the model has other several advantages, including high reproducibility, ease of manipulation, low cost, and the capability of conducting disease regression studies by withdrawal of diet feeding. However, few reports have aimed to demonstrate the chronology of pathological changes, focusing on the cellular crosstalk in the liver using this model. The current study is the first to address the chronology of liver architecture and function, and the behaviour of liver cells, such as KCs, hepatocytes, BECs, LSECs, and HSCs, in this model. Moreover, macroscopic and serum analyses showed that DDC intoxication induces hepatomegaly and cholestasis (Supplementary Figs S2 and S4a). Furthermore, our histological analysis demonstrated the progression of cholestasis in a time-dependent fashion: hepatocyte necrosis from day 1 of feeding, hepatic regeneration from day 3, the peak of inflammation at day 7, and the transition to the chronic phase at day 14 (Figs 1, 7 and S13). In line with these pathological findings, our immunohistochemistry, qRT-PCR, and SEM analyses showed that abnormal activation of each type of liver cell occurred concurrently during the time course: KCs, HSCs, and BECs became activated at day 3, whereas LSECs were activated at day 7 of feeding (Figs 1c, 2, 4, S6a,c and S7a,b).

The current study is the first to demonstrate that ultrastructural changes of various liver cell types are associated with the progression of cholangiopathy (Figs 5a, 6, S7, S11 and S12). In addition, activation of each type of liver cell was directly observed using SEM and TEM, i.e. secretion of collagen fibres by myofibroblasts derived from HSCs, the ductular reaction of proliferative BECs with inter-epithelial neutrophil infiltration, “capillarisation”, the lack of LSEC fenestration and organised basement membranes, and hepatocyte organelle injury such as mitochondrial dysfunction, ER stress, and cytoskeleton dysregulation. Our SEM results also indicated the infiltration of a high number of macrophages in the large central veins in the DDC-induced cholangiopathy (Figs 5b and S10). It is unclear why macrophages predominantly accumulated in the large central veins following DDC intoxication. However, the macrophages in the large central veins were probably derived from monocytes, rather than resident KCs, because monocytes display higher proliferative activity than KCs after liver injuries^17^. Further studies combining the specific inhibition of infiltration of bone marrow-derived monocytes, such as in Mcp1 knockout mice, and the DDC-induced cholangiopathy model could clarify the origin of the main macrophage population that accumulated in the large central veins.

To clarify the precise roles of KCs in the progression and regression phases of cholangiopathy, we depleted KCs by clodronate liposome administration in the DDC diet model. We propose that KCs play a central role in the pathogenesis of cholangiopathy (Fig. 8). A previous study using an acute acetaminophen-induced liver injury model, reported that KCs played beneficial roles in the progression phase, in contrast to our results^4^. These contradictory results between the two studies might be due to differences in the roles of KCs between the acute and chronic phases of liver disease^5^. Macrophages are highly plastic cells whose phenotype is rapidly altered as local signals change, indicating their diverse functions based on the nature and magnitude of the insult^18^. Duffield *et al*. first noted that KCs can have both detrimental and beneficial roles in liver diseases, and suggested that macrophage plasticity is a key determinant for maintaining the balance between their detrimental and beneficial effects^19^. The current study also demonstrated that the effects of KCs were opposite between the progression and regression phases of the disease (Figs 3 and 7). Further, based on our data and other studies, we also propose that phenotypic changes in other liver cells occur subsequent to those in KCs and that the cellular crosstalk between KCs and other liver cells may also contribute to the equilibrium between the adverse and favourable effects^15^. Gieseck *et al*. demonstrated that the IL-13 signaling pathway serves as a key driver for recovery from liver inflammation and fibrosis, through the immunomodulatory roles of macrophages and the interactions between macrophages and other cells^20^. However, Mosser *et al*. demonstrated that no clear demarcation exists between the different proinflammatory (classical, M1) and anti-inflammatory (alternative, including regulatory and wound-healing, M2) macrophage phenotypes^21^. They suggested that macrophage phenotypes should be regarded as a “spectrum” of populations based on their various functions, depending on different phases and different liver injuries. Further studies are needed to better understand how macrophage heterogeneity affects other liver cells in chronic liver diseases.

Our findings have several clinical implications. First, the administration of drugs as specific inhibitors, by focusing on the polarisation of classically activated macrophages in the early phase of chronic liver diseases, could lead to better patient prognosis. Second, for patients in the regression phase of chronic liver disease, the administration of drugs that enhance the activity of regulatory and/or wound-healing macrophages could promote recovery by antagonizing the excessive proinflammatory, cholestatic, and fibrogenic responses. Furthermore, the development of novel biomarkers that can differentiate between macrophage phenotypes could help to appropriately distinguish the disease state and aid early intervention.

The major limitations of our study are that we did not evaluate the effects of specific macrophage phenotypes, the extent to which the bone marrow-derived macrophages and resident KCs contribute to the progression and regression of the disease, and the novel molecular pathways associated with the cellular crosstalk between KCs and other liver cells. Future studies using cellular tracking and/or genetic techniques to focus on these unresolved issues should be performed to gain a better understanding of the role of macrophages in liver diseases^22^.

In conclusion, our results provide new insights into the roles of KCs and the pathophysiological mechanisms underlying the progression of cholangiopathy. KCs can play opposite roles during the progression and regression phases of liver diseases. Moreover, the interactions of KCs with other liver cells may represent one of the main mechanisms for maintaining the balance between the injury-inducing and tissue-repairing tasks. Therefore, macrophages could be a promising target for the establishment of novel therapeutic, diagnostic, and preventive strategies for chronic liver diseases.

## Materials and Methods

### Animals

Male C57BL/6 mice (CLEA Japan Inc., Tokyo, Japan) (8 weeks old, weighing 20–25 g) were used for all experiments. We chose the DDC diet model to study the roles of KCs in cholangiopathy, considering that it well mimics the human primary sclerosing cholangitis, and reproduces the gradual progression of the disease. Moreover, the ease of manipulation and of conducting disease regression studies by withdrawing the diet make this model attractive^23^. To evaluate the time course of histopathological changes in DDC-induced cholangiopathy, mice were fed a 0.1% DDC diet for 1, 3, 7, or 14 days (Supplementary Fig. S1a). To evaluate the roles of macrophages in the early phases of cholangiopathy, mice were repeatedly intraperitoneally injected with clodronate- or PBS-liposomes during the 7 days of DDC or standard diet feeding, from 2 days before the treatment until 1 day before sacrifice (Supplementary Fig. S1b). For the regression experiment, another group of animals was fed a 0.1% DDC diet for 14 days and allowed to recover on a standard diet for an additional 7 days; from 2 days prior to DDC withdrawal until 1 day before sacrifice, they were repeatedly injected with clodronate- or PBS-liposomes (Supplementary Fig. S1c). The control mice were fed a standard diet. To assess the toxicity of clodronate, survival data for the group of mice that were repeatedly injected with clodronate- or PBS-liposomes during the 7-day treatment were analysed using the Kaplan-Meier method and the log-rank test.

The experimental animals were sacrificed at each of the indicated time points. Blood was collected by cardiac puncture after a 2-h fast. For electron microscopy analyses, the mice were perfused for 1 min with saline and fixed by transcardial perfusion (approximately 100 mmHg) with 2% glutaraldehyde and 4% paraformaldehyde. At least three mice were treated and analysed per time period and treatment. All mice were housed individually in cages under specific pathogen-free conditions with food and water ad libitum. There was no difference in food intake between the groups (data not shown).

All experimental protocols were approved by the Animal Care and Use Committee of Kyoto University Graduate School of Medicine, and were performed according to the criteria outlined in the Guide for the Care and Use of Laboratory Animals, prepared by the National Academy of Sciences, as published by the National Institutes of Health (NIH)^24^.

### Serum biochemical analyses

Serum samples were stored at −80 °C until the analyses could be performed. The AST, alanine aminotransferase, alkaline phosphatase, direct-bilirubin, indirect-bilirubin, and bile acid levels were analysed using a Hitachi 7180 analyser (Hitachi, Tokyo, Japan).

### Histopathology

Formalin-fixed paraffin-embedded 4-μm-thick sections were cut and stained with haematoxylin and eosin, Azan, periodic acid Schiff, and reticulin. Frozen sections were cut to 6 μm. The grading and staging of liver histological changes were evaluated as previously described^16,25^. Briefly, the grading of portal inflammation and interface hepatitis and the staging of fibrosis were performed according to the modified Ishak system using a 20× objective. The quantification of spotty necrotic cells, mitotic cells, and neutrophils was performed in random chosen fields, with 10×, 40×, and 60× objectives, respectively. The reticulin fibrosis score was graded as follows: (0), none; (1), slight; (2), mild; (3) intermediate; (4) severe black-stained reticulin fibrosis. For the quantification of glycogen storage, fibrosis, and porphyrin deposition, data were analysed using ImageJ software (US National Institutes of Health, Bethesda, MD, USA), in random chosen fields with 10×, 20×, and 10× objectives, respectively. The histological features of the specimens were independently assessed by an experienced pathologist (MM) and an attending pathologist (HK) in a blinded fashion, and a consensus diagnosis was obtained for each sample. To determine the inter-observer reproducibility of each feature, unweighted kappa coefficients were calculated. The results showed good inter-observer agreement, as demonstrated by kappa coefficients of 0.63 (good) for portal inflammation, 0.71 (good) for interface hepatitis, 0.78 (good) for fibrosis staging of Azan staining, and 0.65 (good) for fibrosis staging of reticulin staining.

### Immunohistochemistry studies

Immunohistochemical staining of the specimens was performed as previously described^26^. Briefly, antigen retrieval was performed in a pressure cooker by boiling in 10 mM citrate buffer (pH 6.0), followed by washing with phosphate-buffered saline. Subsequently, the endogenous peroxidase was quenched with 3% H_2_O_2_ for 10 min at room temperature. After rinsing, the slides were treated overnight at 4 °C with a negative control reagent or the following optimally diluted primary antibodies: F4/80 (rat monoclonal; 1:200; Abcam, ab6640), pan-cytokeratin (pan-CK; mouse monoclonal; 1:40; Abcam, ab27988), and α-smooth muscle actin (αSMA; rabbit polyclonal; 1:400; Abcam, ab5694). Next, the slides were incubated with a horseradish peroxidase-conjugated secondary antibody (goat polyclonal; prediluted; MBL, Nagoya, Japan). Diaminobenzidine or Permanent Red were used as chromogens, followed by counterstaining with haematoxylin. For quantitative assessment of protein expression, the staining of each immunohistochemical specimen was captured in randomly selected fields with a 20× objective and quantitated using ImageJ software.

### Quantitative reverse transcription polymerase chain reaction

Total RNA was extracted from liver tissues using TRIzol (Invitrogen, Carlsbad, CA, USA). cDNA was synthesised from total RNA using SuperScript III reverse transcriptase (Invitrogen). Real-time PCR was performed using FastStart SYBR Green Master mix (Roche Diagnostics, Basel, Switzerland) and a Rotor-Gene Q (Qiagen, Venlo, Netherlands) instrument. The primer pairs are shown in Supplementary Table S1. The relative target gene expression was normalised to the expression of GAPDH mRNA.

### Transmission and scanning electron microscopy analyses

Perfusion-fixed livers were cut into 1-mm sections for transmission electron microscopy (TEM) and into 2-mm sections for scanning electron microscopy (SEM). These sections were immersion-fixed in 2% glutaraldehyde at 4 °C for 2 hours. The specimens for TEM were extensively washed with phosphate-buffered saline, postfixed in 1% osmium tetroxide, dehydrated in a graded series of ethanol, and embedded in Epon. Ultra-thin sections (80 nm) were cut on an Ultra microtome EM UC6 (Leica, Vienna, Austria), stained with 1% uranyl acetate, counterstained using the Reynolds method, and examined on an H-7650 electron microscope (Hitachi). SEM specimens were postfixed in 1% osmium tetroxide, dehydrated in a graded series of ethanol, and dried. Subsequently, the sections were coated with a thin layer of platinum/palladium and visualised under an S-4700 electron microscope (Hitachi). To evaluate the severity of capillarisation, the proportion of open space area around the LSECs (porosity) was measured as a percentage in 15 randomly selected fields at a magnification of 10,000× in at least three animals per group, using ImageJ.

### Statistical analysis

Data are reported as the arithmetic means ± SEM. All data were analysed using SPSS version 20.0 software (SPSS, Tokyo, Japan). Statistical significance was determined using Student’s *t*-test or the Mann-Whitney *U* test with the Bonferroni correction for multiple comparisons. For all analyses, *P* < 0.05 was considered statistically significant.

## Electronic supplementary material

Supplementary information
