# Supplementary material for: Pivotal roles of Kupffer cells in the progression and regression of DDC-induced chronic cholangiopathy
Source: Sci Rep. 2018 Apr 23;8:6415. doi: 10.1038/s41598-018-24825-x (PMC5913224; doi:10.1038/s41598-018-24825-x)
Supplement: Supplementary file 1 — Supplementary information [file 41598_2018_24825_MOESM1_ESM.pdf]

Pivotal roles of Kupffer cells in the progression and regression of DDC-induced chronic cholangiopathy

Leila Jemail, Masashi Miyao*, Hirokazu Kotani, Chihiro Kawai, Hirozo Minami, Hitoshi Abiru, and Keiji Tamaki

Department of Forensic Medicine, Kyoto University Graduate School of Medicine, Kyoto, Japan

***Corresponding author:**

Masashi Miyao, M.D.

Department of Forensic Medicine, Kyoto University Graduate School of Medicine, Yoshida-Konoe-cho, Sakyo-ku, Kyoto 606-8501, Japan

Telephone: +81 75 753 4474; Fax: +81 75 761 9591;

E-mail: miyaom@fp.med.kyoto-u.ac.jp

Supplementary Table S1

Supplementary Table S1. RT-qPCR primers for analysis

Gene	Direction	Sequence
F4/80	Forward	CTCTGTGGTCCCACCTTCAT
F4/80	Reverse	GATGGCCAAGGATCTGAAAA
Mcp1	Forward	CTTCTGGGCCTGCTGTTCA
Mcp1	Reverse	CCAGCCTACTCATTGGGATCA
Il6	Forward	TAGTCCTTCCTACCCCAATTTCC
Il6	Reverse	TTGGTCCTTAGCCACTCCTTC
Tnf-α	Forward	CCCTCACACTCAGATCATCTTCT
Tnf-α	Reverse	GCTACGACGTGGGCTACAG
αSMA	Forward	CCAGAGCAAGAGAGGGATCCT
αSMA	Reverse	TGTCGTCCCAGTTGGTGATG
Timp1	Forward	GCCCTTCGCATGGACATTTA
Timp1	Reverse	CCCCGATCTGCGATGATG
Col1α1	Forward	CACGGCTGTGTGCGATGA
Col1α1	Reverse	TCGCCCTCCCGTCTTTG
Tgf-β	Forward	TGGAGCAACATGTGGAACTC
Tgf-β	Reverse	TGCCGTACAACTCCAGTGAC
Gapdh	Forward	AGGTCGGTGTGAACGGATTTG
Gapdh	Reverse	TGTAGACCATGTAGTTGAGGTCA

Supplementary Figure S1

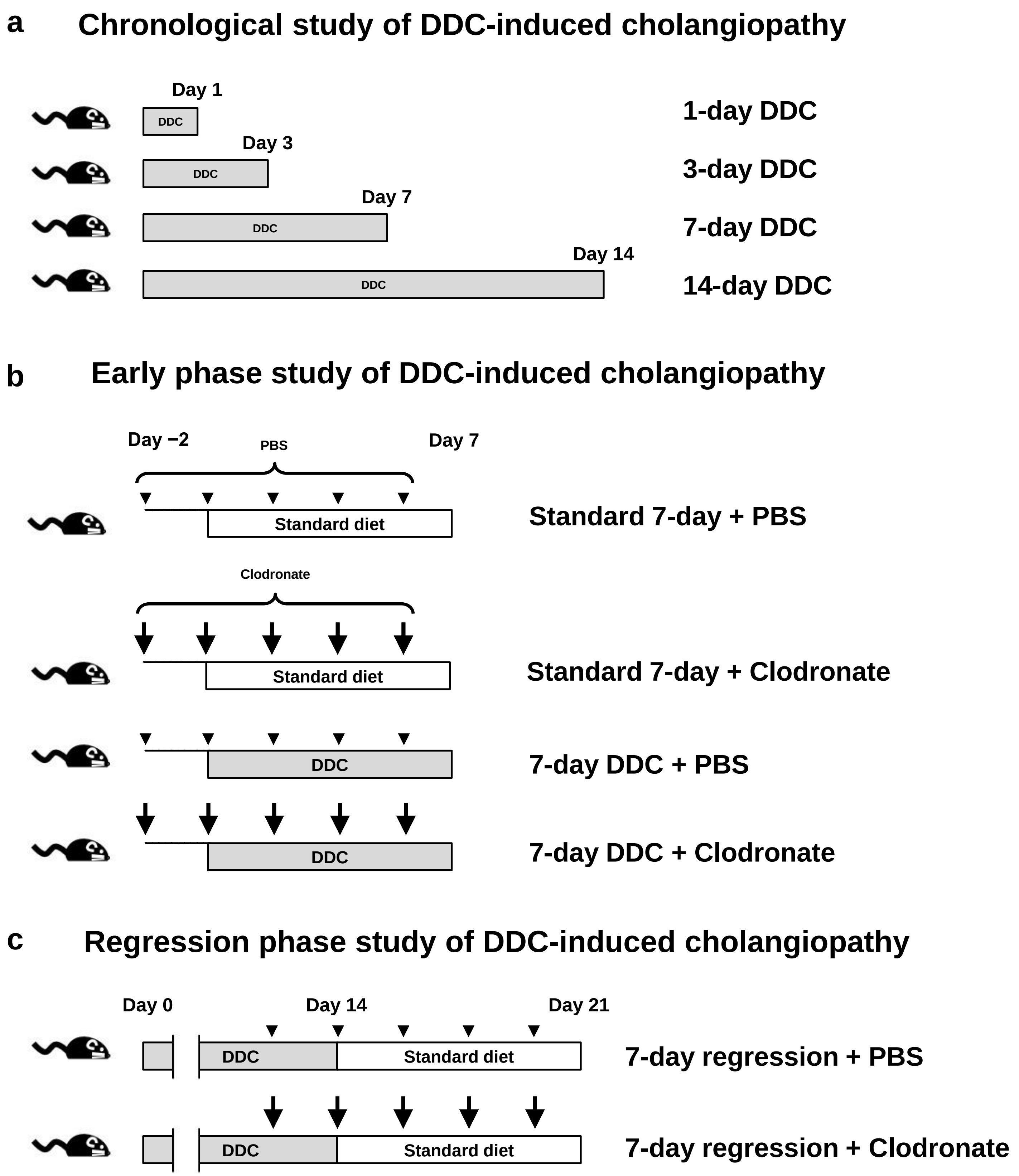

Supplementary Figure S1. (a) Protocol for the chronological study of 3,5-diethoxycarbonyl-1,4-dihydrocollidine (DDC)-induced cholangiopathy. Mice were fed a 0.1% DDC diet for 1, 3, 7, and 14 days. (b) Protocol for early phase study. Mice were fed a standard or a 0.1% DDC diet with repeated intraperitoneal injections of PBS- or clodronate-liposomes from 2 days before feeding until 1 day before sacrifice. (c) Protocol for regression phase study. Mice were fed a 0.1% DDC diet and allowed to recover on a standard diet for an additional 7 days, with last 7 days of repeated PBS- or clodronate-liposome injections.

Supplementary Figure S2

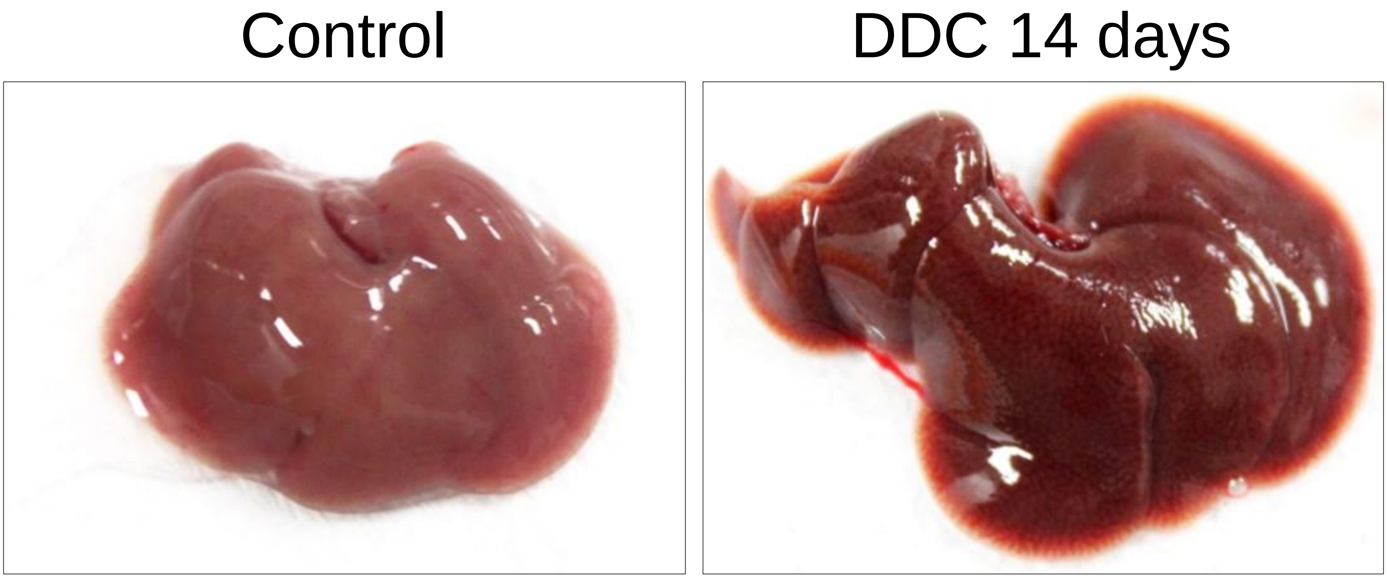

Supplementary Figure S2. Macroscopic photograph of control and 14-day 3,5-diethoxycarbonyl-1,4-dihydrocollidine (DDC)-fed mice. The 14-day DDC-fed mouse shows apparent hepatomegaly and a brownish color change. Six animals were used in each group.

Supplementary Figure S3

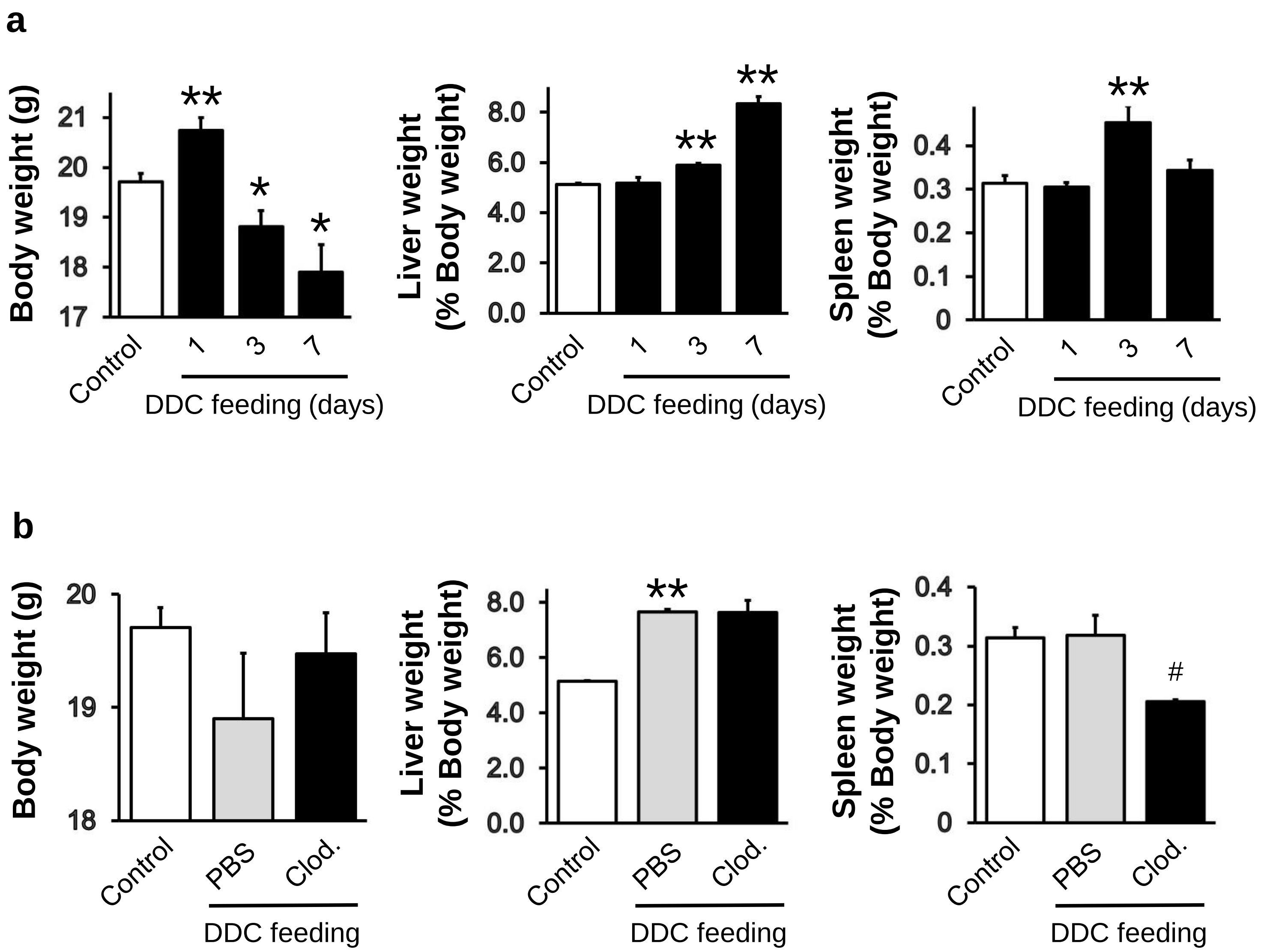

Supplementary Figure S3. (a) Body weight, liver/body weight ratio, and spleen/body weight ratio in control (white boxes) and 3,5-diethoxycarbonyl-1,4-dihydrocollidine (DDC)-fed mice (black boxes) after 1, 3, and 7 days. Data are presented as means ± SEM. *P <0.05 compared with controls. **P <0.01 compared with the controls. (b) Body weight, liver/body weight ratio, and spleen/body weight ratio in control (white boxes), PBS-injected (gray boxes) and clodronate-injected (black boxes), 7-day DDC-fed mice. Data are presented as means ± SEM. **P <0.01 compared with controls. $^{\#}P$ <0.05 compared with PBS-injected mice. Six animals were used in each group.

Supplementary Figure S4

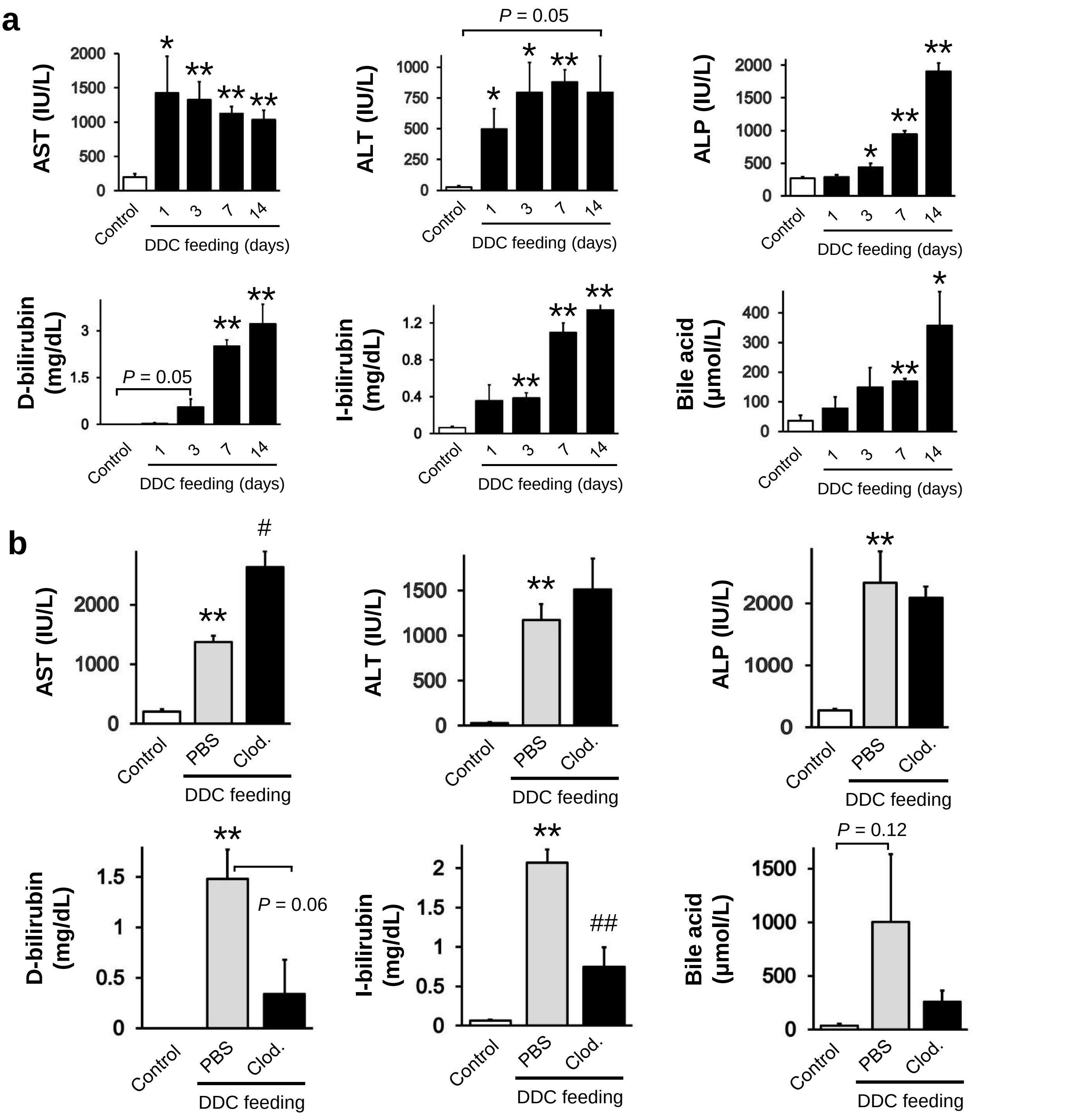

Supplementary Figure S4. (a) Serum levels of aspartate aminotransferase (AST), alanine aminotransferase (ALT), alkaline phosphatase (ALP), direct bilirubin (D-bilirubin), indirect bilirubin (I-bilirubin), and bile acid in control mice (white boxes) and 3,5-diethoxycarbonyl-1,4-dihydrocollidine (DDC)-fed mice (black boxes) after 1, 3, 7, and 14 days. **P* <0.05 compared with controls; ***P* <0.01 compared with controls. (b) Serum levels of AST, ALT, ALP, D-bilirubin, I-bilirubin, and bile acid in control (white boxes), PBS-injected (gray boxes) and clodronate (Clod.)–injected (black boxes), 7-day DDC-fed mice. ***P* <0.01 compared with controls; #*P* <0.05 compared with PBS-injected mice; ##*P* <0.01 compared with PBS-injected mice. Five to six animals were used in each group.

Supplementary Figure S5

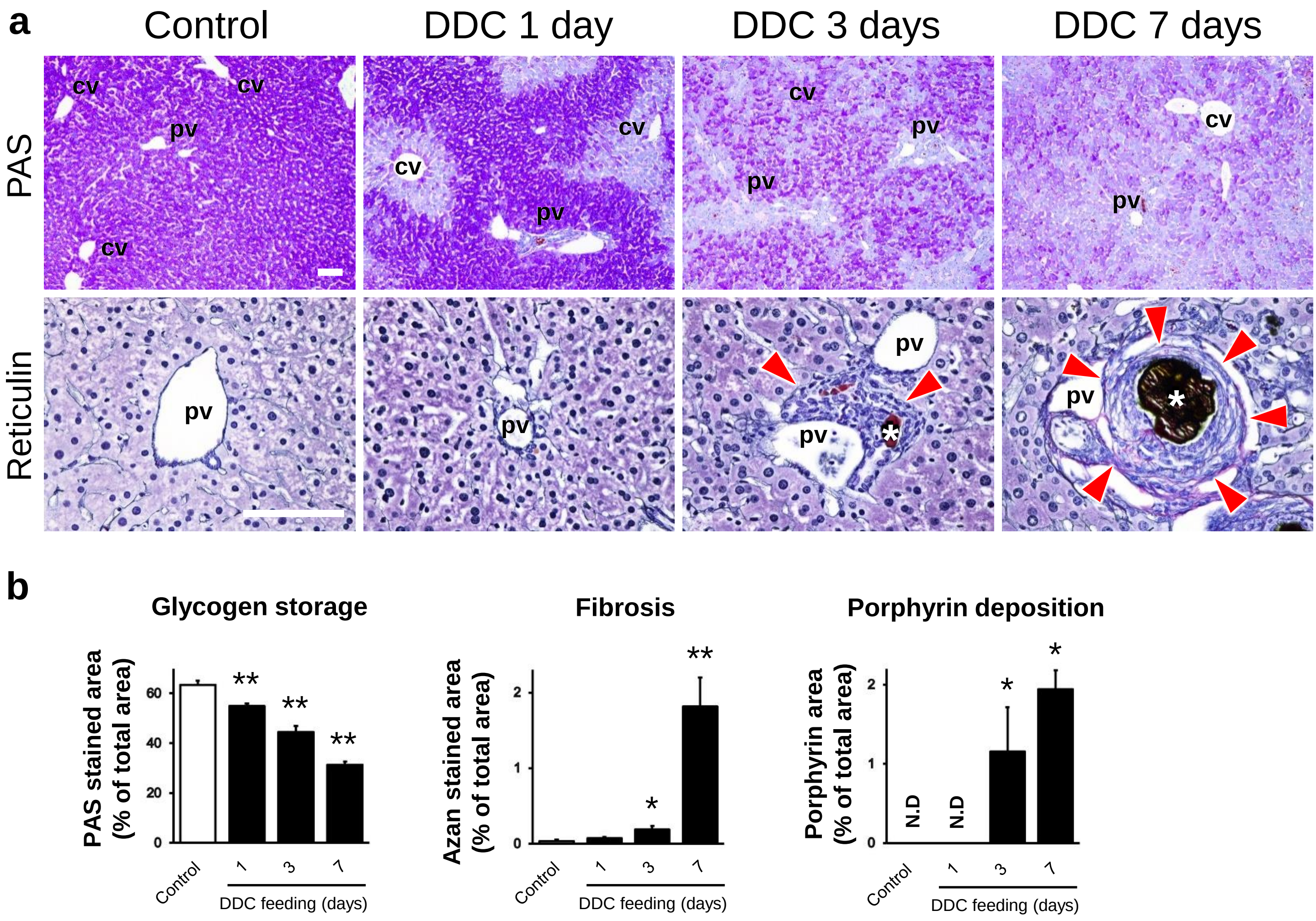

Supplementary Figure S5. (a) Histopathological images of livers from control and 3,5-diethoxycarbonyl-1,4-dihydrocollidine (DDC)-fed mice after 1, 3, and 7 days. Periodic acid Schiff (PAS) staining (top row) and reticulin staining (bottom row) are shown. Bars = 100 μm. Red arrowheads in the reticulin staining sections highlight the deposition of immature collagen fibres in portal triads. pv, portal vein; cv, central vein. (b) Quantitative densitometric analysis of PAS-stained area (glycogen storage area), blue-stained area for Azan staining (fibrosis area), and brown-stained area for H&E staining (porphyrin deposition area) from livers of control and DDC-fed mice after 1, 3, and 7 days. Data are presented as means ± SEM. *P <0.05 compared to controls. **P <0.01 compared to controls. N.D, not detected. Six animals were used in each group.

Supplementary Figure S6

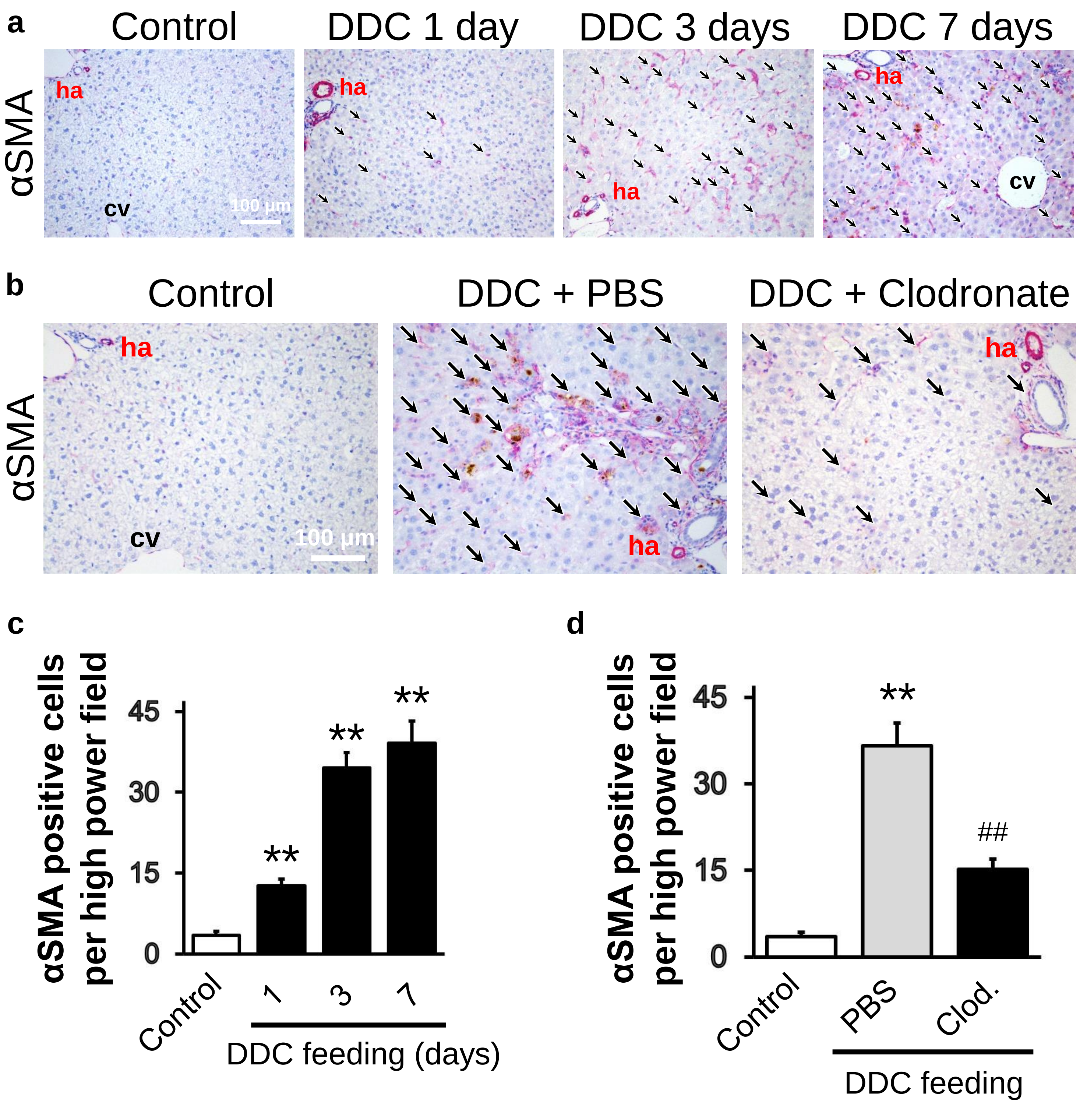

Supplementary Figure S6. (a, b) Immunohistochemical staining for α smooth muscle actin (αSMA) in control, 3,5-diethoxycarbonyl-1,4-dihydrocollidine (DDC)-fed mice after 1, 3, and 7 days, and PBS-injected and clodronate-injected, 7-day DDC-fed mice. Smooth muscles of hepatic arteries (ha) show the internal positive control-stained areas. Arrows indicate the positive cells for the antibody. cv, central vein. (c, d) Quantitative analyses for the antibody-positive cells per high power field in the each mice. Data are presented as means ± SEM. $^{**}P$ <0.01 compared with each control; $^{\#\#}P$ <0.01 compared with the PBS-injected mice. Three animals were used in each group.

Supplementary Figure S7

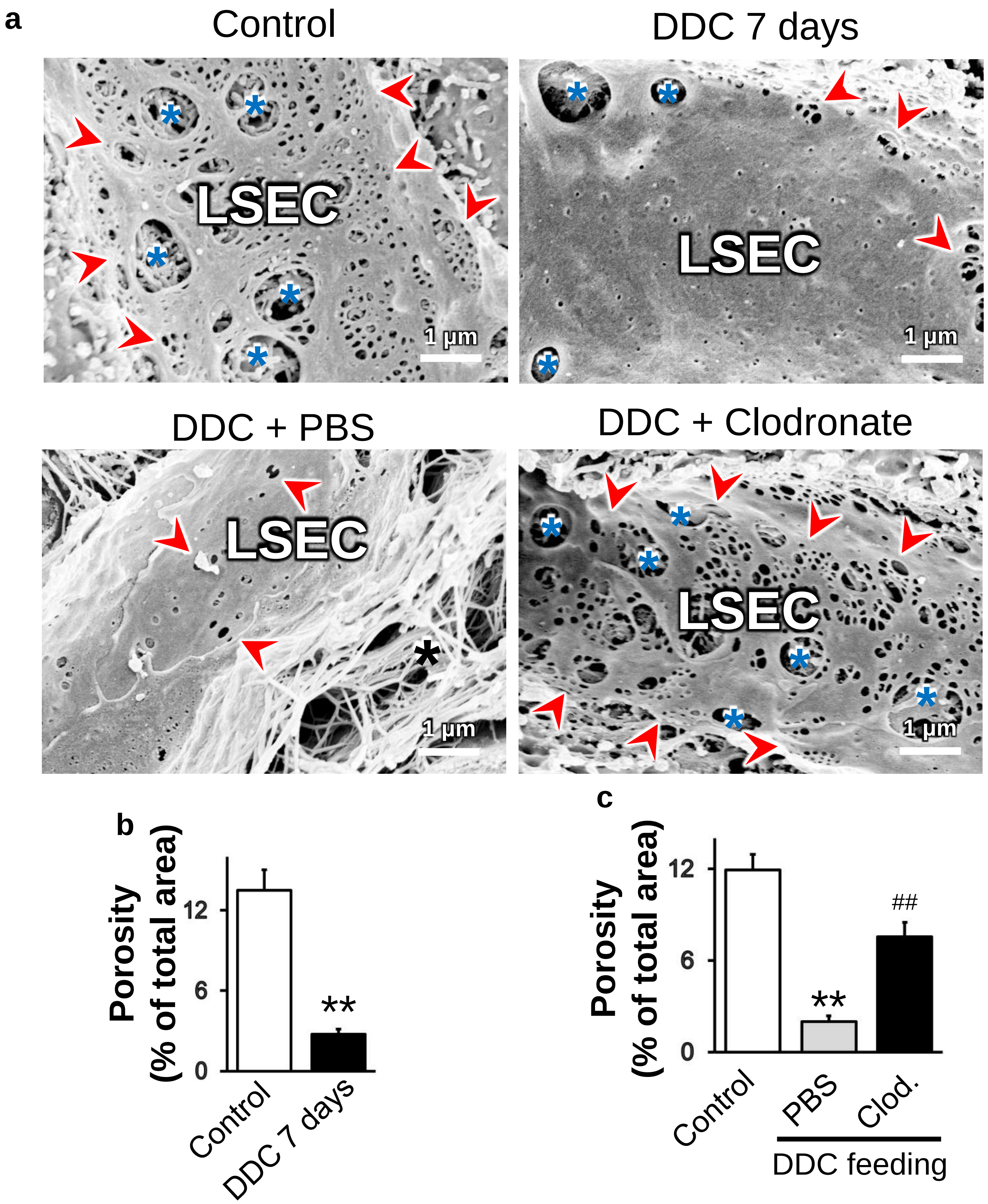

Supplementary Figure S7. (a) Scanning electron microscopy photographs of control, 7-day 3,5-diethoxycarbonyl-1,4-dihydrocollidine (DDC)-fed, PBS-injected and clodronate (Clod.)-injected 7-day DDC-fed mice. Red arrowheads highlight fenestrae in liver sinusoidal endothelial cells (LSECs). Blue asterisks highlight gaps in LSECs. (b) Porosity in control (white box) and DDC-fed mice (black box). (c) Porosity in control (white box), PBS-injected (gray box) and clodronate-injected (black box), DDC-fed mice. $^{**}P < 0.01$ compared to controls, $^{\#\#}P < 0.01$ compared to PBS injected, DDC-fed mice. Three animals were used in each group.

Supplementary Figure S8

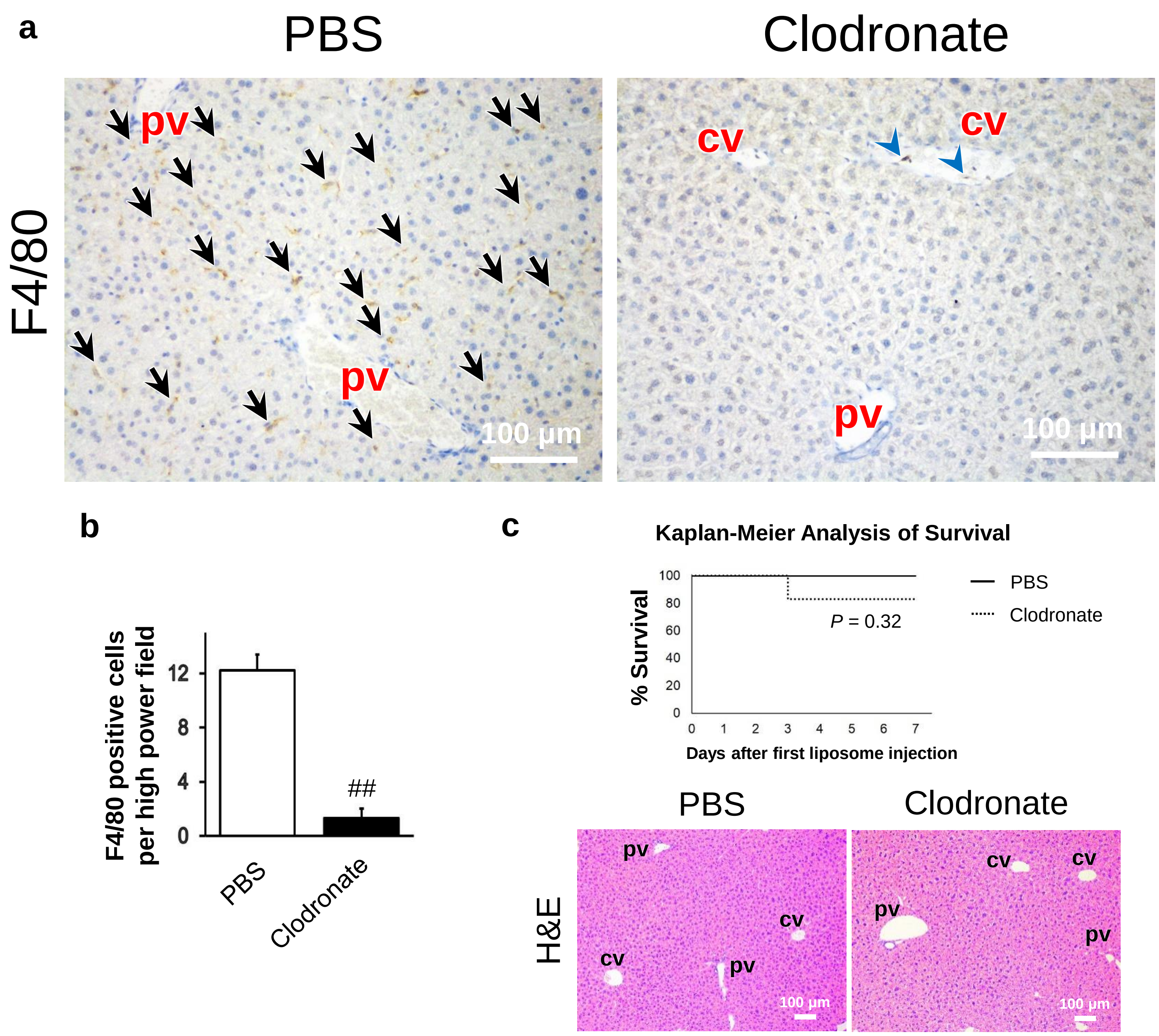

Supplementary Figure S8. (a) Immunohistochemical staining for F4/80 in livers from PBS-injected and clodronate-injected mice. Black arrows indicate Kupffer cells. Blue arrowheads indicate monocytes in the central vein. cv, central vein; pv, portal vein. (b) Quantitative analysis of the numbers of F4/80 positive cells in PBS-injected and clodronate-injected mice. Data are presented as means ± SEM. $^{##}P$ <0.01 compared with PBS-injected mice. Three animals were used in each group. (c) Kaplan-Meier survival analysis and H&E staining of livers from PBS-injected and clodronate-injected mice. No significant changes are observed in the survival analysis and histology between the two groups. cv, central vein; pv, portal vein. Six animals were used in each group.

Supplementary Figure S9

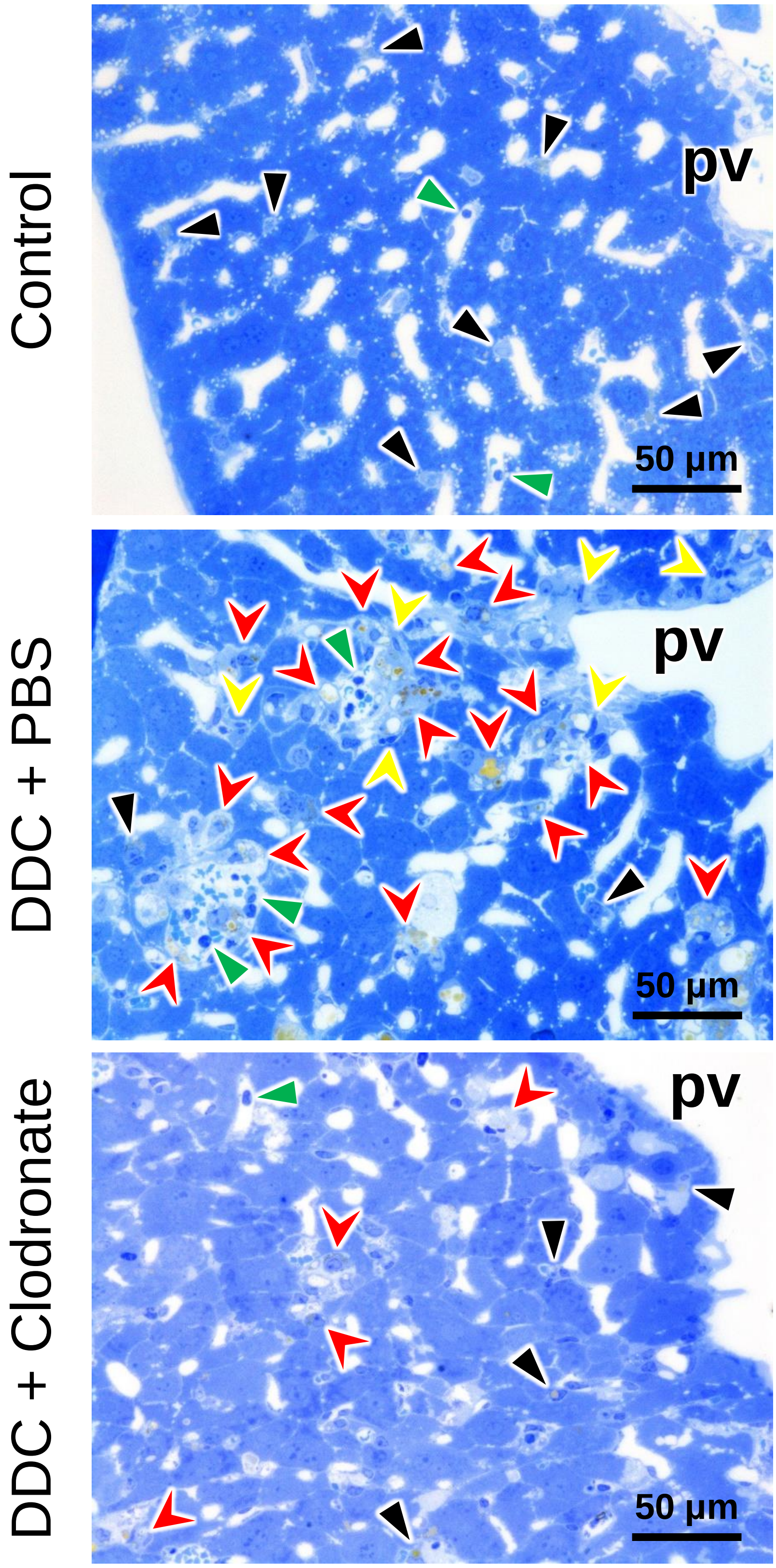

Supplementary Figure S9. Toluidine blue staining of semi-thin liver sections for control, PBS-injected, and clodronate (Clod.)-injected, 7-day 3,5-diethoxycarbonyl-1,4-dihydrocollidine (DDC)-fed mice. Green arrowheads indicate Kupffer cells or recruited monocytes. Black arrowheads indicate quiescent hepatic stellate cells (HSC; lipid storing cells; Ito cells). Red arrowheads indicate activated (phagocytosed) macrophages. Yellow arrowheads indicate spindle shaped myofibroblasts (activated HSCs). pv, portal vein. Three animals were used in each group.

Supplementary Figure S10

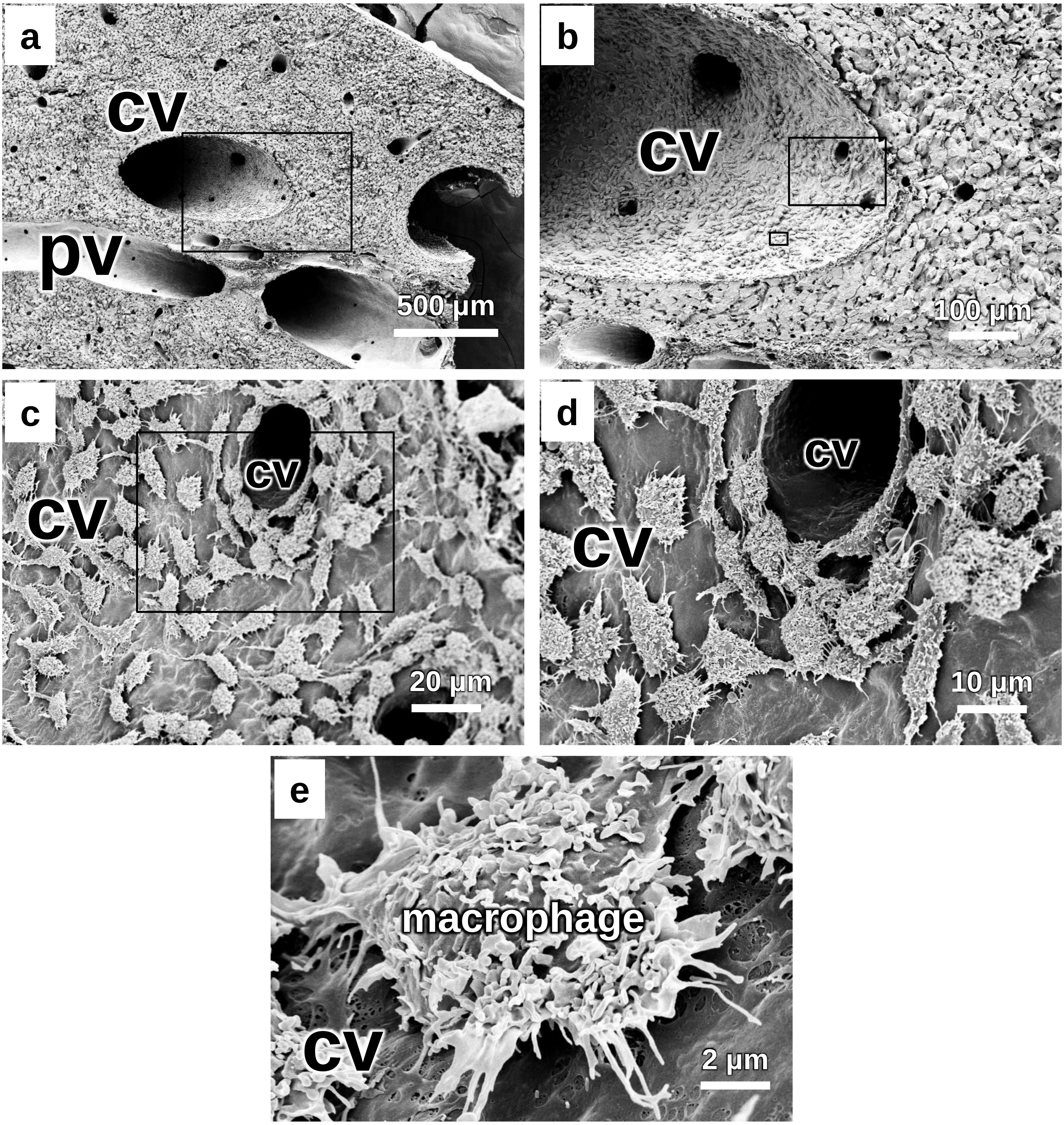

Supplementary Figure S10. Scanning electron microscopy photographs of 7-day 3,5-diethoxycarbonyl-1,4-dihydrocollidine (DDC)-fed mice. (a) The lowest magnification image (30x). A boxed area highlights (b). (b) The second lowest magnification image (100x). The big boxed area highlights (c). The small boxed area highlights (e). (c) A middle magnification image (500x). The boxed area highlights (d). (d) The second highest magnification image (1k x). (e) The highest magnification image (5k x). cv, central vein; pv, portal vein. Three animals were used in each group.

Supplementary Figure S11

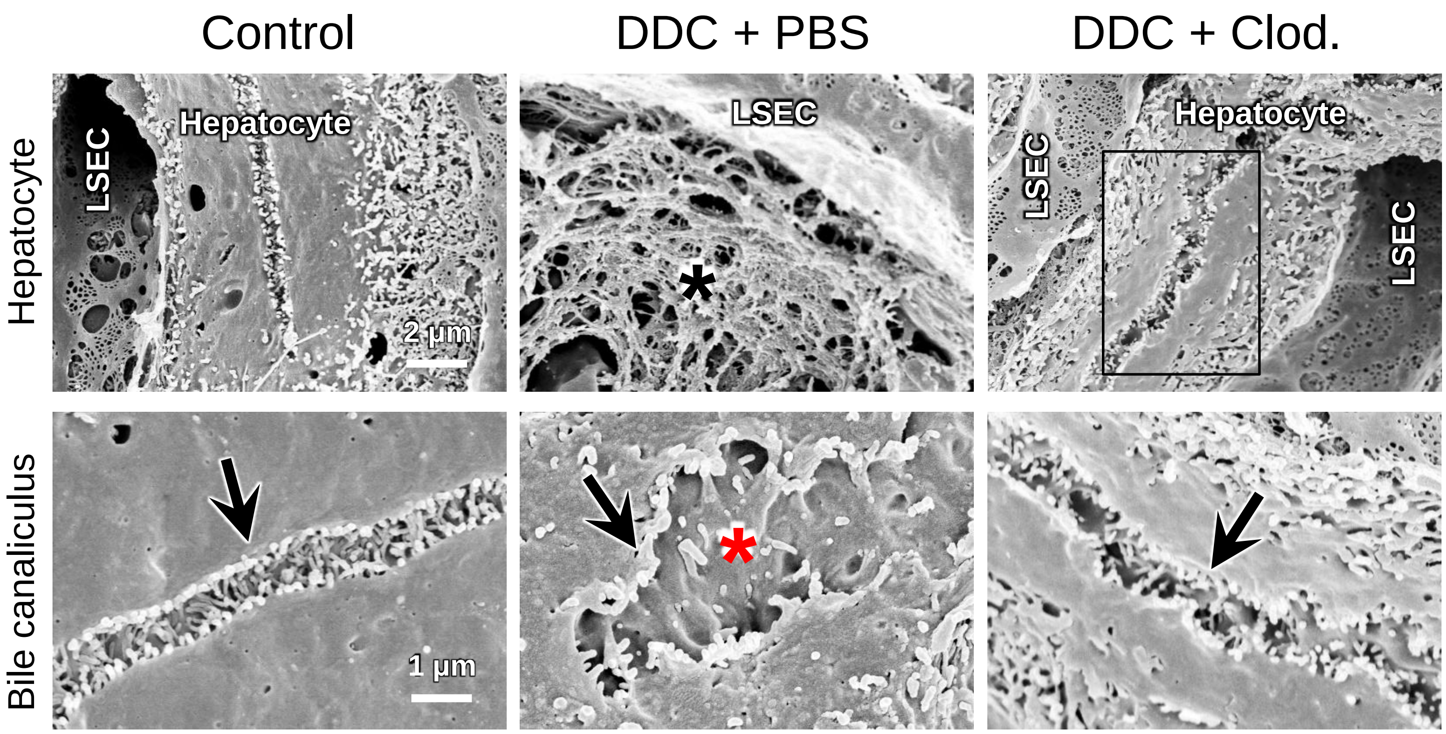

Supplementary Figure S11. Scanning electron microscopy photographs of control, PBS-injected and clodronate-injected, 7-day 3,5-diethoxycarbonyl-1,4-dihydrocollidine (DDC)-fed mice. Upper panels show hepatocytes. A black asterisk shows necrotic hepatocytes with accumulation of collagen fibers. A liver sinusoidal endothelial cell (LSEC) of PBS-injected, DDC-fed mice shows loss of fenestrae. Lower panels show bile canaliculi in hepatocytes. A boxed area highlights the bottom right panel. Black arrows highlight the bile canaliculi. A red asterisk highlights a tortuous and elongated bile canaliculus with loss of microvilli. Three animals were used in each group.

Supplementary Figure S12

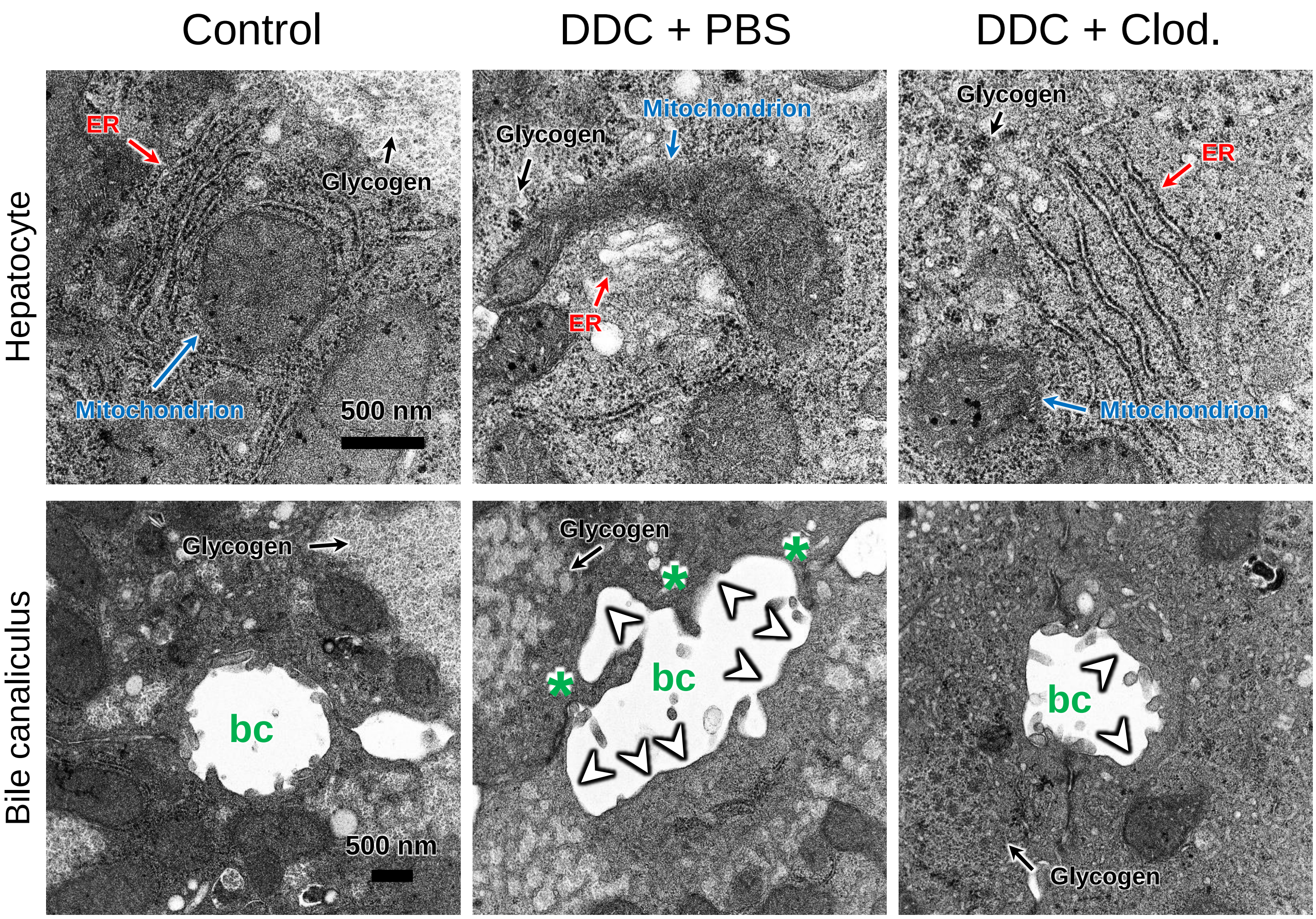

Supplementary Figure S12. Transmission electron microscopy photographs of control, PBS injected and clodronate injected, 7-day 3,5-diethoxycarbonyl-1,4-dihydrocollidine (DDC)-fed mice. Upper panels show organelles in the hepatocytes. A mitochondrion of the PBS-injected DDC-fed mouse shows a deformed shape, with disarranged cristae. Endoplasmic reticula (ER) in the mouse show elongation with loss of ribosomes. The number of glycogen particles in the mouse were severely reduced, especially in β particles (rosette-type particles). Bottom panels show bile canaliculi. Green asterisks highlight bleb formations in plasma membrane of the hepatocyte, likely due to cytoskeletal dysfunction. White arrowheads highlight loss of microvilli in bile canaliculi. bc, bile canaliculi; ER, endoplasmic reticula. Three animals were used in each group.

Supplementary Figure S13

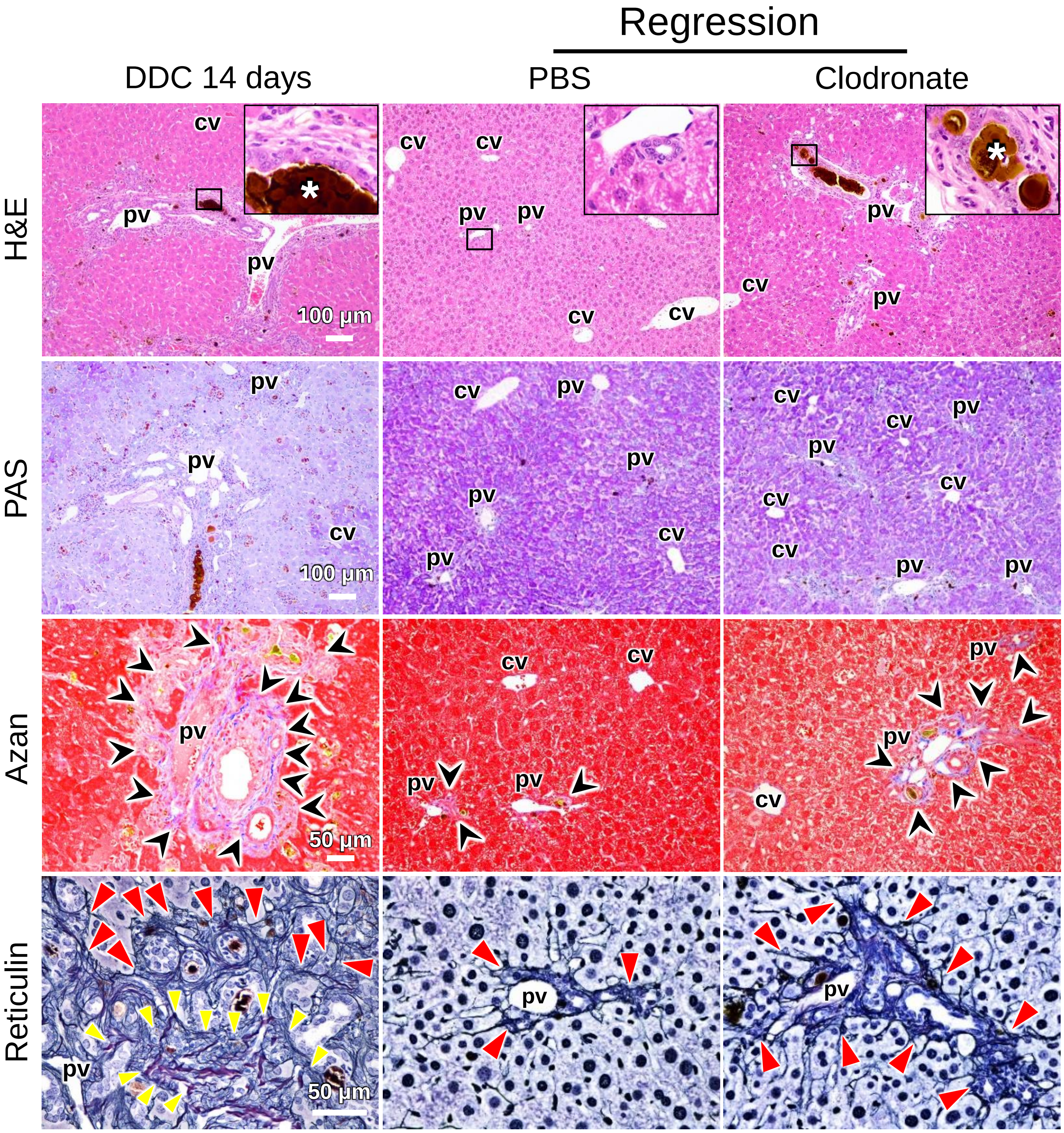

Supplementary Figure S13. Histopathological images of livers from 14-day 3,5-diethoxycarbonyl-1,4-dihydrocollidine (DDC)-fed, PBS-injected and clodronate-injected, 7-day regression mice (14 days of DDC feeding, followed by an additional 7 days of standard diet). Hematoxylin and eosin (H&E) staining, Periodic acid Schiff (PAS) staining, Azan staining, and reticulin staining are shown. Insets of the H&E staining represent high magnification images of black boxed areas in each group. Black arrowheads in the Azan staining sections highlight blue stained fibrotic areas. Red and yellow arrowheads in the reticulin staining sections highlight the deposition of immature and mature collagen fibers in portal triads, respectively.cv, central vein; pv, portal vein. Six animals were used in control group, and five animals were used in PBS-injected and Clod.-injected, 7-day regression groups.
